# KIF5B and Nup358 Cooperatively Mediate the Nuclear Import of HIV-1 during Infection

**DOI:** 10.1371/journal.ppat.1005700

**Published:** 2016-06-21

**Authors:** Adarsh Dharan, Sarah Talley, Abhishek Tripathi, João I. Mamede, Matthias Majetschak, Thomas J. Hope, Edward M. Campbell

**Affiliations:** 1 Department of Microbiology and Immunology, Stritch School of Medicine, Loyola University, Chicago, Illinois, United States of America; 2 Integrative Cell Biology Program, Stritch School of Medicine, Loyola University, Chicago, Illinois, United States of America; 3 Burn and Shock Trauma Research Institute, Department of Surgery, Stritch School of Medicine, Loyola University, Chicago, Illinois, United States of America; 4 Department of Cell and Molecular Biology, Feinberg School of Medicine, Northwestern University, Chicago, Illinois, United States of America; Duke University Medical Center, UNITED STATES

## Abstract

Following envelope mediated fusion, the HIV-1 core is released into the cytoplasm of the target cell and undergoes a series of trafficking and replicative steps that result in the nuclear import of the viral genome, which ultimately leads to the integration of the proviral DNA into the host cell genome. Previous studies have found that disruption of microtubules, or depletion of dynein or kinesin motors, perturb the normal uncoating and trafficking of the viral genome. Here, we show that the Kinesin-1 motor, KIF5B, induces a relocalization of the nuclear pore component Nup358 into the cytoplasm during HIV-1 infection. This relocalization of NUP358 is dependent on HIV-1 capsid, and NUP358 directly associates with viral cores following cytoplasmic translocation. This interaction between NUP358 and the HIV-1 core is dependent on multiple capsid binding surfaces, as this association is not observed following infection with capsid mutants in which a conserved hydrophobic binding pocket (N74D) or the cyclophilin A binding loop (P90A) is disrupted. KIF5B knockdown also prevents the nuclear entry and infection by HIV-1, but does not exert a similar effect on the N74D or P90A capsid mutants which do not rely on Nup358 for nuclear import. Finally, we observe that the relocalization of Nup358 in response to CA is dependent on cleavage protein and polyadenylation factor 6 (CPSF6), but independent of cyclophilin A. Collectively, these observations identify a previously unappreciated role for KIF5B in mediating the Nup358 dependent nuclear import of the viral genome during infection.

## Author Summary

Fusion of viral and target cell membranes releases the HIV-1 viral capsid, which houses the viral RNA and proteins necessary for viral reverse transcription and integration, into the cytoplasm of target cells. To complete infection, the viral capsid must ultimately traffic to the nucleus and undergo a process known as uncoating to allow the nuclear import of the viral genome into the nucleus, where it subsequently integrates into the genome of the target cell. Here, we show that the concerted actions of microtubule motor KIF5B and the nuclear pore component Nup358 cooperatively facilitate the uncoating and nuclear import of the viral genome. Moreover, we also identify the determinants in the viral capsid protein, which forms the viral capsid core, that are required for KIF5B dependent nuclear entry. These studies reveal a novel role for the microtubule motor KIF5B in the nuclear import of the viral genome and reveal potential intervention targets for therapeutic intervention.

## Introduction

Human Immunodeficiency Virus Type-1 (HIV-1), like all primate lentiviruses, possesses the ability to infect non-dividing cells. The ability to infect non-dividing cells is conveyed by the viral capsid (CA) protein which makes up the viral core that houses the viral genome [1,2,3]. CA has important functions during the early stages of HIV infection. Specifically, it acts to shield the viral genome from cytoplasmic sensors capable of inhibiting infection and activating innate immune signaling pathways[4,5,6,7]. The ability to protect the viral genome from host factors in the cytoplasm and also mediate the nuclear import of the viral genome is complicated by the dimensions of the viral core, which at ~120nm x 60 nm [8,9], significantly exceeds the size limitation of nuclear pore cargoes, which is ~39 nm [10,11]. These findings collectively suggest that core disassembly, known as uncoating, must be properly regulated so that the viral genome can be delivered to the nucleus while keeping the genome shielded from host factors in the cytoplasm. CA must therefore interact with numerous host factors to ensure that these functions are performed in a spatiotemporally appropriate fashion.

Genome wide screens for host factors required for replication identified numerous proteins associated with the nuclear import machinery of the cell, including the nuclear pore complex (NPC) proteins Nup358 and Nup153 [12,13,14]. In the context of the nuclear pore, Nup358 forms a basket on the cytoplasmic side of the NPC, while Nup153 serves a similar function on the opposite side of the NPC (reviewed in [15]) Functional studies demonstrate that the virus preferentially relies on NUP358 and Nup153 to enter the nucleus of non-dividing cells [16,17,18,19,20,21,22]. In the case of Nup153, the ability to support HIV-1 infection maps to the viral CA protein [21], and structural studies have found that the phenylalanine/glycine repeats (FG repeats) present on Nup153 are able to bind a conserved “pocket” on assembled CA[22,23,24,25]. This binding pocket, which is formed by inter-molecular association of N-terminal and C-terminal domains in adjacent CA proteins in the CA hexamer, is also the binding site for numerous cellular factors and antiviral compounds, including cleavage and polyadenylation specificity factor 6 (CPSF6) [20,23,24]. In contrast to Nup153, the role of Nup358 in HIV-1 infection remains less clear. Nup358 has a cyclophilin (Cyp) homology domain, which is capable of binding CA at the conserved Cyp binding loop present on CA, and has been reported to induce isomerization of the peptide bond at P90 to facilitate infection and core disassembly (uncoating)[18,26]. However, other studies have shown that the dependence of HIV-1 infection on Nup358 does not require the Cyp homology domain [27], leaving the mechanism by which Nup358 interacts with the viral ribonucleoprotein complex and facilitates infection unclear.

We and others have recently observed that microtubule motors dynein and the kinesin-1 motor KIF5B are required for HIV-1 uncoating and infection[28,29,30]. Specifically, uncoating in the cytoplasm was inhibited by microtubule disruption or by depletion of dynein heavy chain or KIF5B[28,30]. However, these studies did not determine the mechanism by which microtubule trafficking was coupled to the disassembly of the viral core.

Here, we show that depletion of theKinesin-1 motor KIF5B leads to the accumulation of HIV-1 viral cores at the nucleus. During infection, KIF5B mediated anterograde trafficking of viral cores is accompanied by the displacement of Nup358 from the nuclear membrane to the cytoplasm, where it associates with viral cores. Critically, this cytoplasmic trafficking of Nup358 and its association with viral cores is perturbed by mutations which disrupt the hydrophobic binding pocket in assembled CA (N74D) and mutations which disrupt the conserved Cyp binding loop on CA (P90A). The functional relevance of this interaction was demonstrated by the observation that KIF5B depletion inhibits nuclear import of the viral genome in a CA dependent manner, as N74D and P90A CA mutants are not sensitive to KIF5B depletion. These studies provide evidence of a bipartite interaction between CA and Nup358, in which the hydrophobic binding pocket and Cyp binding loop both contribute to this association. These studies demonstrate, for the first time, the determinants required for the interaction of Nup358 and the viral core, and identify a critical role for KIF5B in the nuclear import of HIV-1 during infection.

## Materials and Methods

### Cell lines

HeLa and 293T cells (ATCC) were cultured in Dulbecco’s modified Eagle medium (DMEM) (Cellgro) supplemented with 10% fetal bovine serum (FBS), 1000 U/ml penicillin, 1000 U/ml Streptomycin and 10 μg/ml Ciprofloxacin Hydrochloride. HeLa TZM-bl cells stably expressing CD4 and CCr5 were obtained through the NIH AIDS Reagent Program, Division of AIDS, NIAID, NIH: TZM-bl from Dr. John C. Kappes, Dr. Xiaoyun Wu and Tranzyme Inc. To generate monocyte derived macrophages (MDM), peripheral blood mononuclear cells (PBMC) were obtained from peripheral blood (Loyola University IRB 208423)immediately after collection by layering over lymphocyte separation medium (Corning) and spinning at 2000rpm for 30min. The PBMC were washed twice in PBS and monocytes positively selected using the EasySep Human CD14 positive selection kit (STEMCELL) following the manufactures protocol. Isolated monocytes were resuspended in RPMI media supplemented with 10% fetal bovine serum (FBS), 1000 U/ml penicillin, 1000 U/ml Streptomycin and 10 μg /ml Ciprofloxacin Hydrochloride. Monocytes were differentiated to macrophages by resuspending in RPMI media containing 50 ng/ml granulocyte macrophage colony-stimulating factor (GM-CSF) and 50 ng/ml macrophage colony-stimulating factor (M-CSF). Cells were plated on culture dishes and differentiated for 8 days before performing experiments.

### Ethics statement

Human blood obtained for this study was de-identified prior to our work. We did not have any interactions with the human subject, or protected information, and therefore no informed consent was required.

### Virus production and synchronized infection

To generate pseudotyped HIV-1, 293T cells seeded in a 15cm dish at 60% confluency were transfected with 8.25 μg pCMV-VSVg and 16.75 μg of R7ΔEnvGFP using polyethylenimine (PEI, MW 25000 Polysciences). HIV-1 virus harboring the HXB2 glycoproteins were generated by transfecting 293T cells on 10cm dish with 7 μgR7ΔEnvGFP and 3 μg HXB2 envelope glycoproteins using PEI.P8.9NDSB is a minimal HIV-1 packaging plasmid for *gag* and *pol* expression and described before [31]. p8.9NDSB wildtype (WT) and capsid mutants N74D and P90A was kindly provided by Jeremy Luban(University of Massachusetts Medical School)[32]. To generate pseudotyped reporter virus, 293T cells seeded on 10cm dish were transfected with 3 ug p8.9NdSB WT or mutants, 2 μg pCMV-VSVg and 5 μg pLVX-GFP (clontech). MLV reporter virus were produced by PEI transfection of 293T cell on 10cm plate with 5 μg of pCigB (generous gift of Dr. Greg Towers), 3 μg of GFP reporter vector [33] and 2 μg of pCMV-VSVg. Gag-integrase-Ruby was kindly provided by Thomas J. Hope (Northwestern University) and virus were produced by PEI transfection of 293T cell on 10cm plate with 5 μgR7ΔEnvGFP, 2.5 μg Gag-intergrase-Ruby and 2.5 μg pCMV-VSVg. Viruses were harvested 48 hours after transfection, spun for 5 minutes at 1200 rpm and filtered through a 0.45 μm filter (Milipore). Synchronized infection was performed as described previously [34]. In brief, cells were spinoculated at 13°C for 2 hours at 1200xg, after which virus containing medium was removed and replaced with 37°C media. Infectivity was measured 48 hours post synchronized infection and percentage of GFP positive cells was determined using BD FACSCanto II cytometer (BD Bioscience). Multiplicity of infection (MOI) was calculated based on the equation: MOI = -lnP(0) where P(0) is the proportion of uninfected cells.

### Antibodies and chemicals

HIV-1 capsid protein p24 was stained using either anti-p24 AG3.0 (mAb from Dr. Jonathan Allan) or 183-H12-5C (HIV-1 p24 hybridoma from Dr. Bruce Chesebro) and were obtained through the NIH AIDS Reagent Program, Division of AIDS, NIAID, NIH. Rabbit polyclonal antibodies against Nup358 (ab64276) and Nup153 (ab84872) were purchased from Abcam. Rabbit anti-KHC to detect kinesin-1 heavy chain (sc-28538) were from Santa Cruz Biotechnology. Rabbit polyclonal to CPSF6 (ab99347) was purchased from Abcam and rabbit polyclonal to cyclophilin A (PA1-025) was purchased from ThermoScientific. Secondary antibodies conjugated to fluorophore for immunofluorescence studies were purchased from Jackson Immunoresearch Laboratories. Cyclosporine A (CsA; Sigma Aldrich) was used at a final concentration of 2.5 μM. DAPI to stain nucleus was obtained from Sigma Aldrich.

### Western blotting

Cell lysates were prepared by lysing cells with NP-40 lysis buffer (100mM Tris pH 8.0, 1% NP-40, 150 mM NaCl) containing protease inhibitor cocktail (Roche) for 10 minutes on ice. Following incubation, lysates were spun down at 13,000 rpm for 10 min and supernatant collected for western blot analysis. In brief, 2x Laemmli sample buffer were added to the lysed sample and incubated at 100°C for 5min. Protein concentration was measured using Pierce BCA protein assay kit (Thermo Scientific) and equal amount of protein was loaded in to an 8% polyacrylamide gel for SDS-polyacrylamide gel electrophoresis (SDS-PAGE). To detect Nup358, proteins were loaded on to a 4–15% gradient gel (Bio-Rad). Upon separation, the proteins were transferred to nitrocellouse membrane (Bio-Rad). Membranes were probed using specific primary antibodies and then probing with secondary antibodies conjugated to Horseradish Peroxidase (HRP) (Thermo Scientific). Antibody complexes were detected using SuperSignal West Femto Chemiluminescent Substrate (Thermo Scientific). Chemiluminescence was detected using the ChemiDoc Imaging System (Bio-Rad).

### RNAi interference

siRNA sequences targeting KIF5B heavy chain [35] and Nup358 [36] has been described before. siRNA targeting human cyclophilin A (sc-105263) and CPSF6 (sc-72990) were obtained from Santa Cruz Biotechnology, Inc. A control siRNA targeting luciferase was purchased from Fisher Scientific. siRNA’s were transfected in to HeLa or HeLa TZM-bl cells plated on 6 well plate using Lipofectamine 2000 (Thermo Fisher) as per the manufactures protocol. A second transfection was performed 24 h later. 72h after the first siRNA transfection, cells were collected and seeded on to 24 well plate for subsequent experiments a day later. Western blotting was performed to monitor knockdown efficiency of the siRNA.

### Microscopy and image acquisition

Z-stack images were collected with a DeltaVision wide field fluorescent microscope (Applied Precision, GE) equipped with a digital camera (CoolSNAP HQ; Photometrics), using a 1.4-numerical aperture 100× objective lens. Excitation light was generated with an Insight SSI solid state illumination module (Applied Precision, GE) and were deconvolved with SoftWoRx deconvolution software (Applied Precision, GE). In any experiment, identical acquisition conditions were used to acquire all images. Following deconvolution, images were analyzed by Imaris 7.6.4 (Bitplane). An algorithm was designed using the surface feature function in Imaris to generate surfaces around signal of interest and the maximum fluorescence intensity associated within these surfaces were measured. The algorithm was applied to all images within the same experiment. For live cell experiments, cells were plated in delta DPG dishes (Thermo Fisher Scientific). Cells were maintained at 5% CO_2_ at 37°C in an environmental chamber on a DeltaVision microscope, and images were captured in a z series on an electron multiplied charge coupled device digital camera (EMCCDCascade 2; Photometrics) and deconvolved using SoftWoRx deconvolution software. Images were acquired every 15 seconds for 10 minutes.

### *In situ* uncoating assay

The *in situ* uncoating assay has been described before [37]. For the assay, fluorescently labeled HIV-1 was generated by transfecting 293T cells on a 25cm dish with 6.25 μg S15-mCherry, 5.25 μg GFP-Vpr, 7.5 μg R7ΔEnvGFP, and 6 μg pCMV-VSVg using PEI. Following fusion, S15-mCherry labeled viral membrane is lost which allows to effectively discriminate between viruses that have non- productively endocytosed by the target cells (S15-mCherry+, GPF-Vpr+) from those that have productively fused into the cytoplasm (S15-mCherry−, GFP-Vpr+). A synchronized infection performed on HeLa cells with these fluorescent labeled virions (Labeling efficiency, 94%). Following spinoculation, media was aspirated and changed to 37°C warm media. Cells were incubated at 37°C and fixed at various time points post infection. Coverslips were then subjected to indirect immunofluorescence as described before [37]to stain for viral capsid protein p24 using the anti-p24 mAb AG3.0 and a Cy5 conjugated secondary antibody (Jackson Immunoresearch) and mounted on glass slides. Images were acquired at 100x magnification using DeltaVision wide field fluorescent microscope. 20 fields acquired per coverslip. After deconvolution, GFP-Vpr viral complexes was identified using the surface function in Imaris (Bitplane) software and the maximal S15-mCherry and p24 signal present within these individual GFP-Vpr generated surfaces was determined. From the large sets of data acquired, the average maximal p24 intensity of fused (S15-mCherry negative) populations of virions was determined.

### Real time PCR

Cells were infected with equal amount of virus as determined through a p24 Elisa kit (Advanced Bioscience laboratories) and RT-PCR was performed to determine the late reverse transcription (Late RT) and 2-LTR products and carried out as described before [38]. GAPDH was used as a housekeeping gene for normalization. Primers for GAPDH were forward, GCACCGTCAAGGCTGAGAAC and reverse, GCCTTCTCCATGGTGGTGAA. Genomic DNA from cells was extracted following the DNeasy Blood and Tissue Kit protocol (Qiagen). DNA concentration was determined and equal amount of DNA was digested with Dpn1 (New England BioLabs) before performing RT-PCR.

### Proximity ligation assay

Duolink proximity ligation assay (PLA) kit was purchased from Sigma and assay performed as described by the manufacturer (Olink Bioscience). In brief, cells grown on coverslips were fixed with 3.7% PFA 3h post synchronized infection. To detect interaction between viral capsid protein p24 and Nup358, cells were permeabilized and blocked in 3% BSA followed by incubation with primary antibodies targeting viral protein p24 (mouse monoclonal) and Nup358 (rabbit polyclonal). After primary staining, coverslip containing cells were washed and incubated (1h, 37°C) with secondary anti-mouse conjugated with minus and anti-rabbit conjugated with plus Duolink II PLA probes. Coverslips were washed again and incubated with ligation-ligase solution (30min, 37°C) followed by washing and subsequent incubation with amplification-polymerase solution (100min, 37°C) containing Duolink II insitu detection reagent Red. Finally, coverslips were washed and mounted with Duolink II mounting medium containing DAPI. Interactions were detected as fluorescent spots (λ _excitation/emission_ 598/634 nm) under a fluorescence microscope.

### Quantification of perinuclear and cytoplasmic protein signal

To determine the amount of p24 or Nup358 signal present in the cytoplasm and around the nucleus, the DAPI staining to detect nuclei along with the masking function in Imaris was utilized. An algorithm was designed using the DAPI channel and surface function to detect the cell nuclei. To detect nuclear or perinuclear signal, all signal outside the nuclei surface generated was masked using the masking tool in Imaris and saved as a separate channel. Then a new algorithm was designed utilizing this new channel and the surface function to detect the p24 or Nup358 signal present around the nucleus. The total sum intensity of these surface masks was calculated to determine the amount of protein present around the nucleus. Similarly, to detect cytoplasmic signal, all signal outside of the nuclear surface mask was determined. The relative fraction of the perinuclear signal was calculated as a percentage of the total signal (perinuclear + cytoplasmic).

### Statistical analysis

GraphPad Prism version 5.00 (GraphPad Software, Inc.) was employed for statistical analysis and to make graph. Statistical significance was assessed using the One-Way or Two-way ANOVA and Bonferroni posttest. P<0.05 was considered significant in our experiments. Data is represented as mean +/- SEM depending on the graph.

## Results

### Nup358 and KIF5B depletion delays HIV-1 uncoating

**Fig 1:**
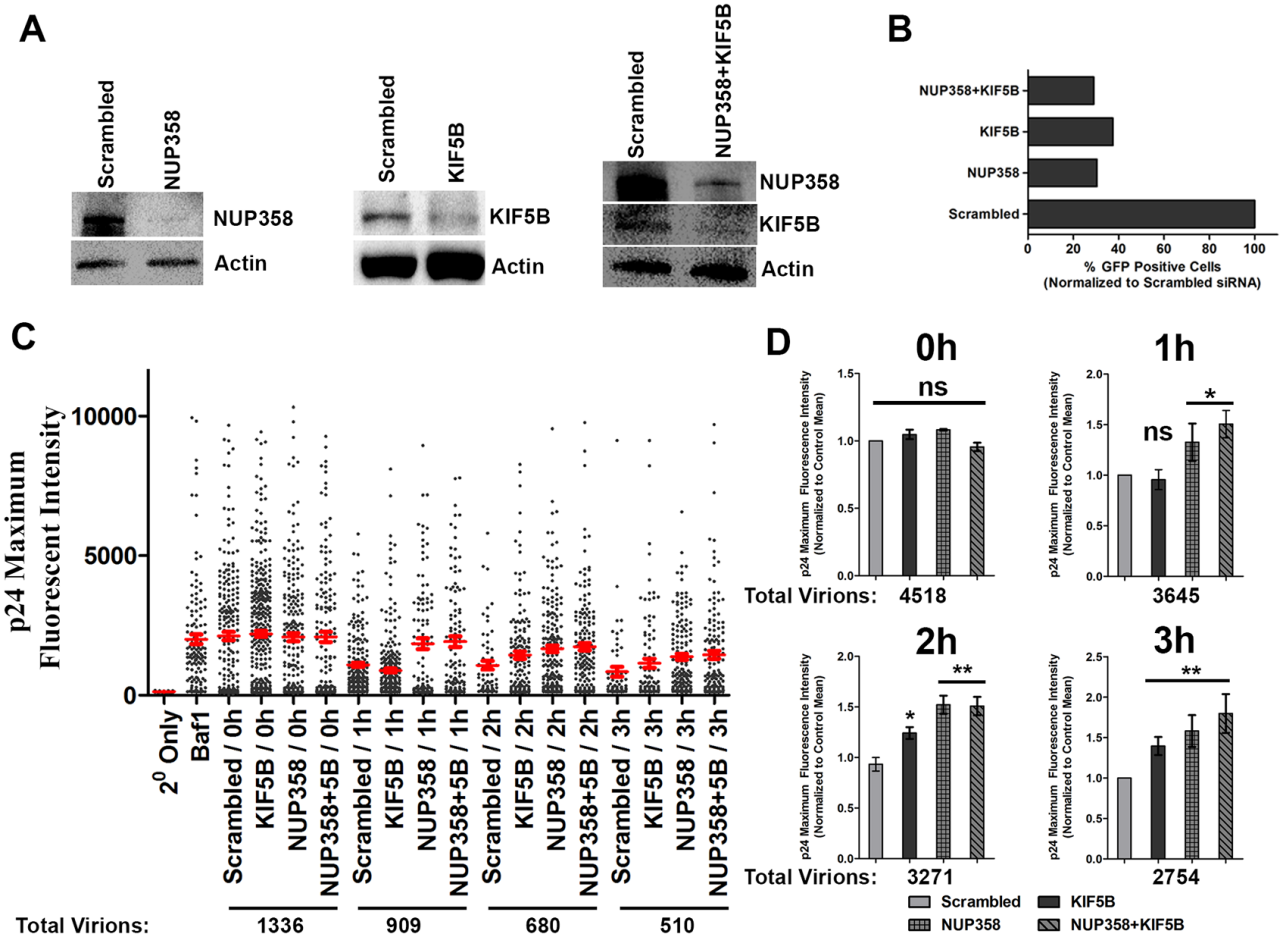
KIF5B and NUP358 facilitate HIV-1 uncoating. **(A)** HeLa cells were transfected with siRNA’s targeting Nup358, KIF5B or both (Nup358+KIF5B). Expression of the indicated proteins were determined by western blot 96h post transfection.**(B)** siRNA treated HeLa cells infected with VSVg-HIV-1 GFP (MOI 0.8) and infectivity measured by determining the percent of GFP positive cells by FACS. **(C)** HeLa cells treated with scrambled, Nup358, KIF5B or Nup358+5B siRNA for 96h were synchronously infected with S15-mCherry/GFP-Vpr VSVg-HIV-1. Cells were fixed at the indicated time point, and the p24 intensities associated with individual virions lacking the S15 membrane label (1–3 hours PI) or all virions (controls and 0 hr points) are shown. Bafilomycin A1 (Baf1), inhibits VSVg mediated fusion and was used as a control in these experiments. Red line is the average p24 intensity measured for all fused viruses at the indicated time point. At least 20 cells were imaged at each time point. Error bars represent SEM.**(D)**Data from three independent experiments, as shown in C, were normalized to the mean p24 intensity observed in control siRNA transfected cell and averaged. **p<0.01, *p<0.05, ns = not significant. Data is representative of three or more independent experiments.

To investigate the role of Nup358 and KIF5B in HIV-1 uncoating, we looked at the uncoating states of individual virions following a synchronized infection in cells depleted of Nup358, KIF5B or both proteins by siRNA using an *in situ* uncoating assay. This assay measures the amount of CA which remains associated with individual viral particles during infection. Viral particles are labeled with GFP-Vpr, and unfused viral particles are removed from analysis using a fluorescent marker of viral membranes (S15-mCherry), allowing the relative amount of p24CA which remains associated with populations of cytoplasmic viral particles to be analyzed [28]. Cells were infected with VSV pseudotyped virus labeled with GFP-Vpr (for labeling viral complexes) and S15-mCherry (to determine fusion state) and fixed at various time points post infection. The amount of p24 associated with individual virions that have productively entered the cells (S15 Negative) was determined by staining with a monoclonal antibody to p24 and measuring the p24 fluorescence intensity using wide field deconvolution microscopy. Western blot analysis of Nup358 and KIF5B showed efficient depletion of each protein at the time of infection (Fig 1A). Depletion of either or both protein was sufficient to substantially inhibit HIV-1 infection (Fig 1B). Following synchronized infection, we observed that depletion of KIF5B delayed HIV-1 uncoating, as measured by p24 staining of individual GFP-Vpr puncta. This effect was apparent in individual experiments (Fig 1C) and in the normalized average of three independent experiments (Fig 1D). Similarly, we observe a significant increase in CA staining in the cytoplasmic virion population in Nup358 knockdown cells (Fig 1C and 1D). A similar effect on uncoating was also observed following depletion of both KIF5B and Nup358, and the effects of depletion of both proteins was similar to the effects observed when either was depleted (Fig 1C and 1D). This delay in uncoating was not due to upstream perturbation of fusion, as a similar percentage of virions had lost their S15 membrane label at the time points examined (S1 Fig). These results demonstrate that Nup358 depletion delays HIV-1 uncoating. The observation that knocking down both Nup358 and KIF5B did not increase the infectivity defect or uncoating defect observed when these proteins were depleted individually is consistent with these proteins acting at a similar step in infection.

### HIV-1 capsids accumulate around the nucleus following KIF5B and Nup358 depletion

**Fig 2:**
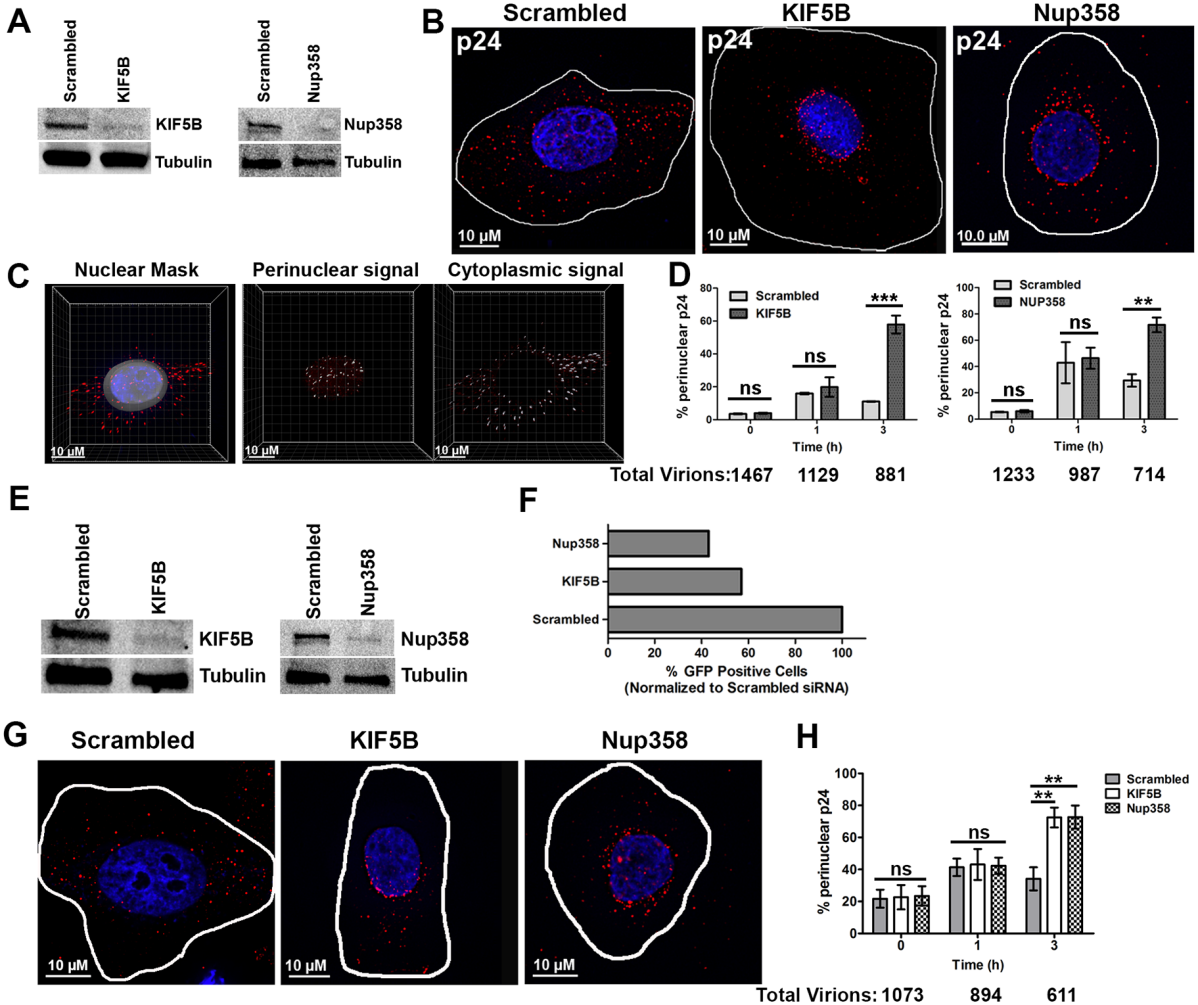
KIF5Band Nup358 depletion lead to the perinuclear accumulation of HIV-1 cores. HeLa cells were transfected with siRNA’s targeting Nup358, KIF5B or a scrambled siRNA sequence.**(A)** Western blot for KIF5B or NUP358 96h following siRNA transfection **(B)**siRNA depleted cells were synchronously infected withVSVg-R7ΔEnvGFP(MOI 0.6). Cells were fixed 0, 1and 3h following a synchronized infection and stained for HIV-1 capsid protein p24 (red) and DAPI (blue) for cell nuclei. A representative image at 3h post infection is depicted.**(C)**Quantification process employed to detect perinuclear and cytoplasmic p24 protein levels. Nuclear mask generated based on the DAPI channel (left image) and perinuclear signal quantified by masking all signal inside (middle image) or outside right image) of the nuclear mask. **(D)**Percentage ofp24 puncta in the perinuclear region in KIF5B and Nup358 depleted cells, quantified as described in (C). TZM-bl cells were transfected with siRNA’s targeting Nup358, KIF5B or a scrambled siRNA sequence.**(E)** Western blot for KIF5B or NUP358 72h following siRNA transfection. siRNA depleted cells were synchronously infected with HXB2-R7ΔEnvGFP(MOI 0.32). **(F)**Infectivity was assessed 48 hours following infection. **(G,H)** Cells were fixed 0, 1and 3h following a synchronized infection and stained for HIV-1 capsid protein p24 (red) and DAPI (blue) for cell nuclei. A representative image at 3h post infection is depicted. **(H)** Percentage ofp24 puncta in the perinuclear region in KIF5B and Nup358 depleted cells, quantified as described in (C). 20 or more cells was analyzed at each time point. Error bars represent the SEM of three experiments. The total number of virions analyzed at each time point is shown below each graph. ***p<0.001, *p<0.05, ns = not significant. Data is representative of three or more independent experiments.

In the experiments described in Fig 1, we also observed that KIF5B depletion and Nup358 depletion (Fig 2A) led to the accumulation of viral cores around the nucleus, rather than the dispersed localization observed three hours following a synchronized infection(Fig 2B).

To quantify this phenotype, we developed a localization assay in which a perinuclear surface mask is generated using DAPI fluorescence. An algorithm which reliably overestimated the boundary of the nuclear DAPI stain was used to measure the fraction of virions within the perinuclear region and in the cytoplasm (Fig 2C). The algorithm developed typically generated a surface mask 2μm from the boundary of the nucleus (DAPI). This method allowed us to quantify the number of p24 puncta around the perinuclear region (inside the perinuclear mask) and in the cytoplasm (outside the nuclear mask) in a large number of cells in an automated and unbiased manner. Using this assay, we observed a significant increase in the amount of capsid localized around the perinuclear region in KIF5B and NUP358 knockdown cells relative to control cells at three hours post-infection (Fig 2D). To ensure that this effect was not due to the mode of viral entry, we performed similar experiments as above in HeLa TZM-bl cells, which express CD4 and coreceptors required for HIV-1 envelope fusion. Depletion ofKIF5B and Nup358 from these cells (Fig 2E) led to a similar decrease in infectivity following infection of these cells with viruses using the HXB2 envelope glycoproteins for entry (Fig 2F). As was observed with VSV-g pseudotyped virions, KIF5B and Nup358 depletion led to a perinuclear accumulation of viral particles around the nucleus, where a more dispersed localization of particles was observed in the control siRNA infected cells (Fig 2G and 2H).

### Nup358 and KIF5B depletion reduce HIV-1 nuclear import and infection

**Fig 3:**
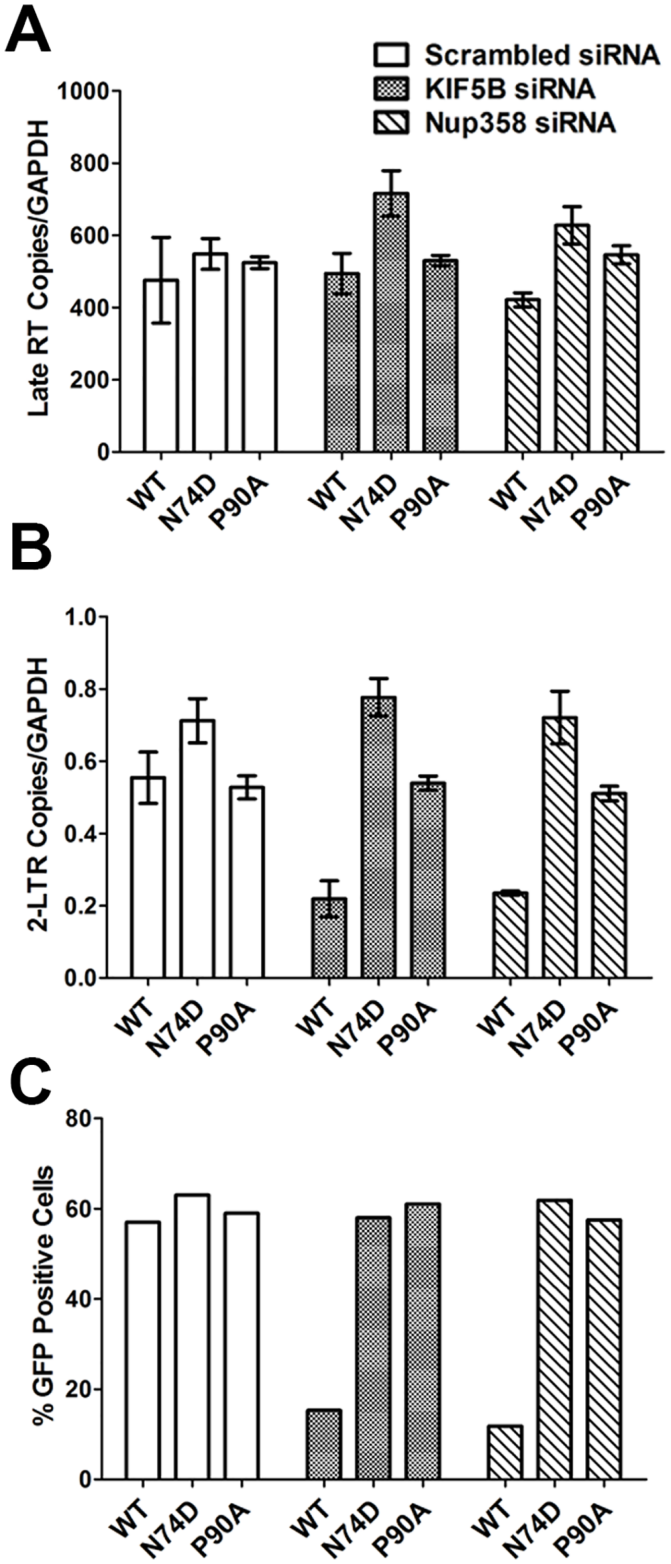
HIV-1 capsid influences the dependence on KIF5B and Nup358 mediated nuclear import during infection. HeLa cells treated with scrambled, Nup358 or KIF5B siRNA for 72h were subjected to synchronized infected with 100ng of VSVg pseudotyped HIV-1 reporter virus bearing either the wildtype (WT) CA or N74D and P90A CA mutants. Cells were collected 24 post infection and real time PCR performed using specific primers to quantify Late RT **(A)** and 2-LTR circle **(B)**. Error bars represent the standard deviation of single samples run in triplicate. **(C)** GFP expression 48h post infection, as measured by flow cytometry of 10,000 cells. The data shown here is representative of three independent experiments.

To understand the functional relevance of KIF5B mediated trafficking of HIV-1 during infection, we measured viral reverse transcription and nuclear import (as measured by 2-LTR formation) products in cells depleted of KIF5B or Nup358 by siRNA using quantitative PCR. Infection with wildtype virus showed no change in the amount of late reverse transcription products following KIF5B or Nup358 depletion (Fig 3A). However, we observed that Nup358 or KIF5B knockdown reduced nuclear import and infection of WT virus (Fig 3B and 3C), consistent with previous reports [18,20,29]. As previously reported, Nup358 depletion did not significantly influence nuclear import or infection by the N74D or P90A mutants [18,20]. Strikingly, we also observed that depletion of KIF5B did not affect nuclear import or infection by the P90A and N74D mutant viruses (Fig 3B and 3C). Collectively, these data demonstrate that WT HIV-1nuclear entry is mediated by a Nup358, KIF5B dependent mechanism, and that nuclear entry of the N74D and P90A virus occur in a Nup358 and KIF5B independent manner.

### HIV-1 infection induces the KIF5B dependent relocalization of Nup358

Given the defective nuclear entry observed following KIF5B and Nup358 knockdown, we examined the ability of HIV to associate with Nup358 during infection. We hypothesized that WT, but not the N74D or P90A mutants, might interact with Nup358 more efficiently during infection. To ensure the specificity of our antibody, we transfected HeLa cells with control siRNA or siRNA targeting Nup358. Nup358 depletion effectively abrogated staining observed using a Nup358 polyclonal antibody, demonstrating that this antibody is not cross-reacting with another cytoplasmic or NPC protein (S2A Fig).

**Fig 4:**
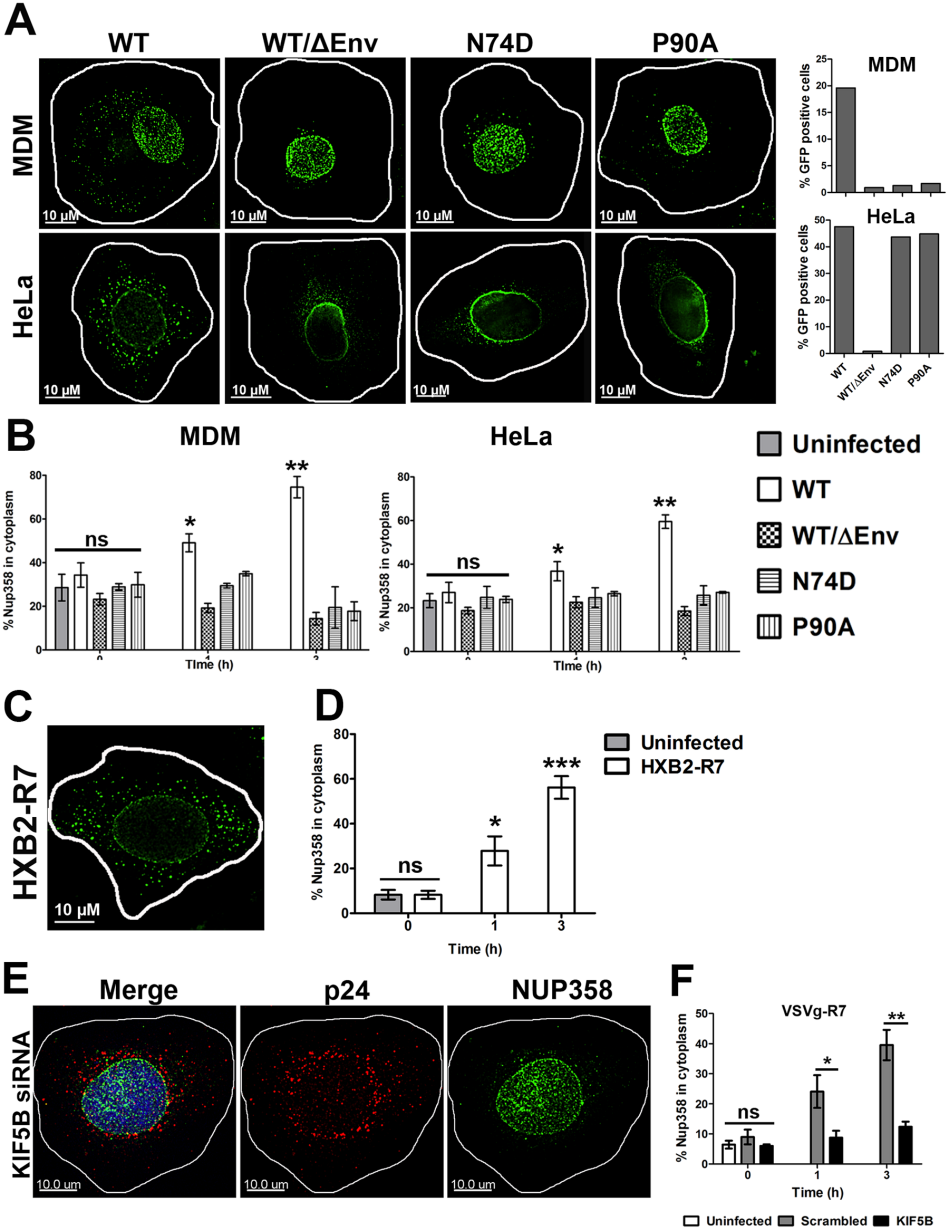
HIV-1 infection induces Nup358 relocalization. **(A,B)** Monocyte derived macrophages (MDM) and HeLa cells were synchronously infected with VSVg pseudotyped HIV-1 reporter virus (MOI 0.3 for MDM and MOI 0.6 for HeLa cells) bearing either the wildtype (WT) CA or N74D and P90A CA mutants. Cells were fixed at 0, 1 or 3h (shown) post infection and stained for Nup358 (green). Infection for each cell type is shown **(B)**The fraction of Nup358 signal in the cytoplasm at the indicated time PI, measured as in 2C.**(C,D)**TZM-Bl cells were synchronously infected with R7ΔEnvGFPpseudotyped with the HXB2 envelope protein (MOI 0.32). Cells were fixed 0, 1 and 3h (shown) post infection and stained for Nup358. **(D)** The fraction of Nup358 signal in the cytoplasm at the indicated time PI.**(E,F)**HeLa cells were transfected with KIF5B specific or scrambled control siRNA and synchronously infected with R7ΔEnvGFPpseudotyped with VSV-g (MOI 0.6)96 hours following siRNA transfection. Cells were fixed 0, 1 and 3h (shown) post infection and stained for Nup358.**(F)**The fraction of Nup358 signal in the cytoplasm at the indicated time PI.20 or more cells were analyzed for each sample. Error bars represent the SEM of three independent experiments. (**p<0.01, *p<0.05, ns = not significant). Data is representative of three or more independent experiments.

In HIV-1 infected monocyte derived macrophages (MDMs), infectivity of the N74D and P90A mutants was attenuated (Fig 4A), while infectivity was not affected in HeLa cells (Fig 3C), as previously reported [5,20]. In both cell types, Nup358 localized to the nuclear membrane and to cytoplasmic puncta three hours after a synchronized infection. In contrast, Nup358 localization following infection with viral particles lacking envelope, or with the N74D or P90A mutants, appears less cytoplasmic and predominated around the nuclear envelope (Fig 4A). Algorithm assisted quantification of Nup358 relocalization confirmed that WT induced the cytoplasmic accumulation of Nup358 in both MDMs and HeLa cells, while virus lacking envelope, or harboring the N74D or P90A mutations, did not (Fig 4A and 4B). Infection with murine leukemia virus (MLV), which cannot infect non-dividing cells, and is not thought to interact with NUP358 during infection, was similarly unable to induce NUP358 relocalization (S2B Fig). This result was not specific for the mode of entry, as HXB2 pseudotyped HIV-1 was able to induce NUP358 redistribution in TZM-bl cells (Fig 4C and 4D). This result was also specific for NUP358, as NUP153 did not colocalize appreciably with HIV-1 capsid (S3C Fig), nor was its localization impacted by HIV-1 infection (S3D Fig).

It is known that Nup358 interacts dynamically with the NPC and Nup358 is also known to be a cargo adapter for KIF5B [39], so we next asked if the cytoplasmic relocalization of Nup358 induced by HIV-1was KIF5B dependent. KIF5B knockdown inhibited the NUP358 relocalization induced by HIV-1 infection (Fig 4E and 4F). Collectively, these data demonstrate that HIV-1 induces NUP358 relocalization during infection in a CA and KIF5B dependent manner.

### Determinants in HIV-1 CA are necessary for Nup358 association with viral cores

**Fig 5:**
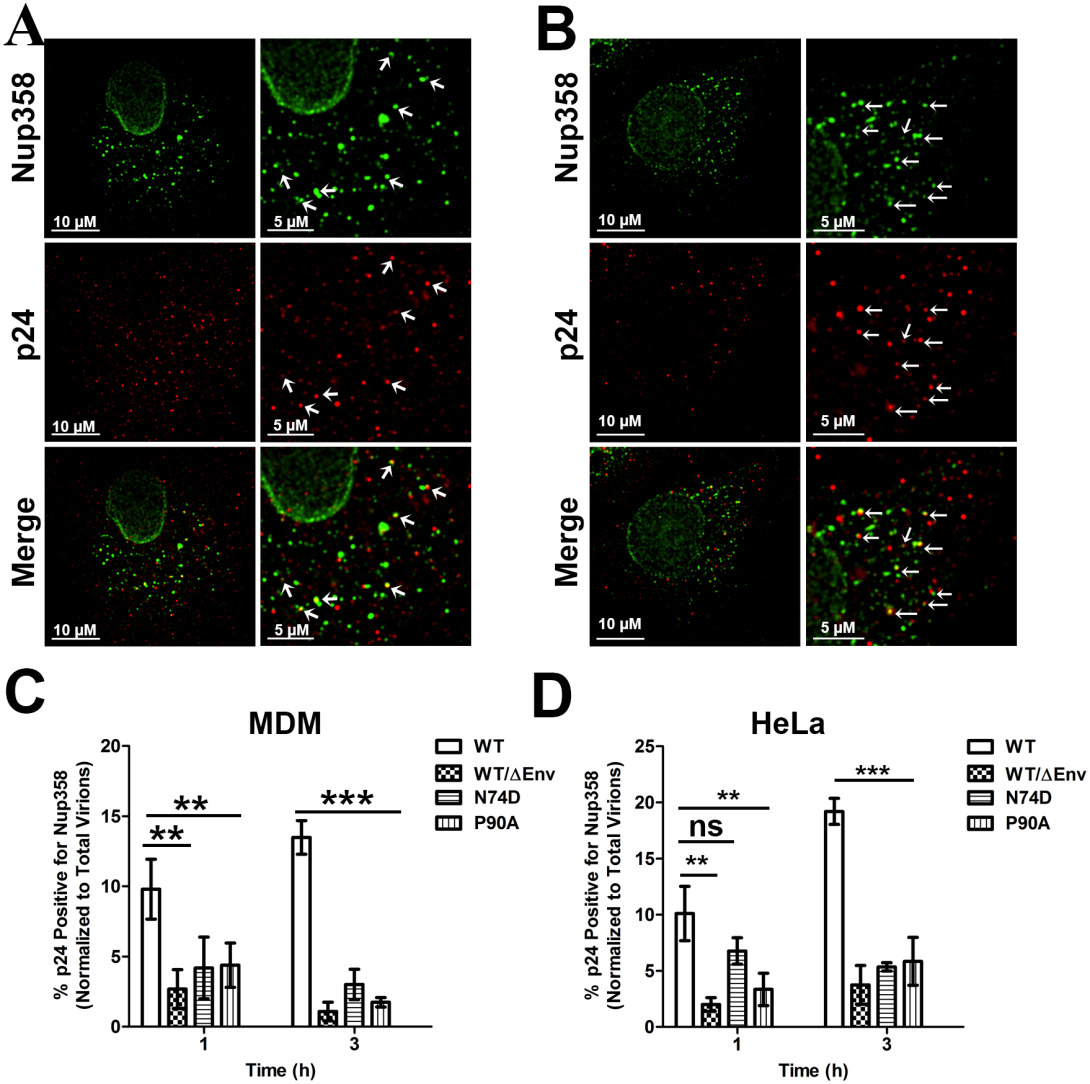
Nup358 and HIV-1 cores colocalize in the cytoplasm during infection. **(A)**MDMs were synchronously infected with HIV-1 GFP pseudotyped with VSV-g bearing either the wildtype (WT) CA or N74D and P90A CA mutants (MOI 0.3).Cells fixed 1 and 3h post infection and stained for viral capsid protein p24 (red) and Nup358 (green). Depicted a representative image at 3h post infection of WT virus (left panel) and an enlarged view of the same image showing colocalization of Nup358 and p24 (right panel and indicated by arrows). **(B)** Similar experiments as A performed on HeLa cells (MOI 0.6). **(C,D)** Quantification of the percent p24 colocalizing with Nup358 in MDM cells **(C)** or HeLa cells **(D)**.20 or more cells were analyzed in each sample. Error bars represent the SEM of three independent experiments. (**p<0.01, *p<0.05, ns = not significant). Data is representative of three or more independent experiments.

We also assessed the degree of colocalization between viral cores and Nup358 following infection of MDMs. Colocalization of Nup358 and HIV-1 cores was readily detectable following WT infection in MDMs (Fig 5A) and HeLa cells(Fig 5B). However, this pattern of colocalization was not apparent after infection with the capsid mutants or WT/ΔEnv (S4 Fig). To quantify the association between Nup358 and HIV-1 cores, we determined the percentage of p24 puncta which were positive for Nup358 in these infections. Three hours following infection of MDMs, a higher percentage of WT viral particles were positive for Nup358 than WT/ΔEnv, N74D or P90A (p<0.001) (Fig 5C). A similar trend was observed 1 hour following infection, although this difference was less significant (p<0.05) (Fig 5C). The degree of colocalization between both the N74D and P90A mutants was similar to the degree of background colocalization between Nup358 and WT/ΔEnv virus (Fig 5C) at both time points. Similar results were also observed in HeLa cells (Fig 5D).

**Fig 6:**
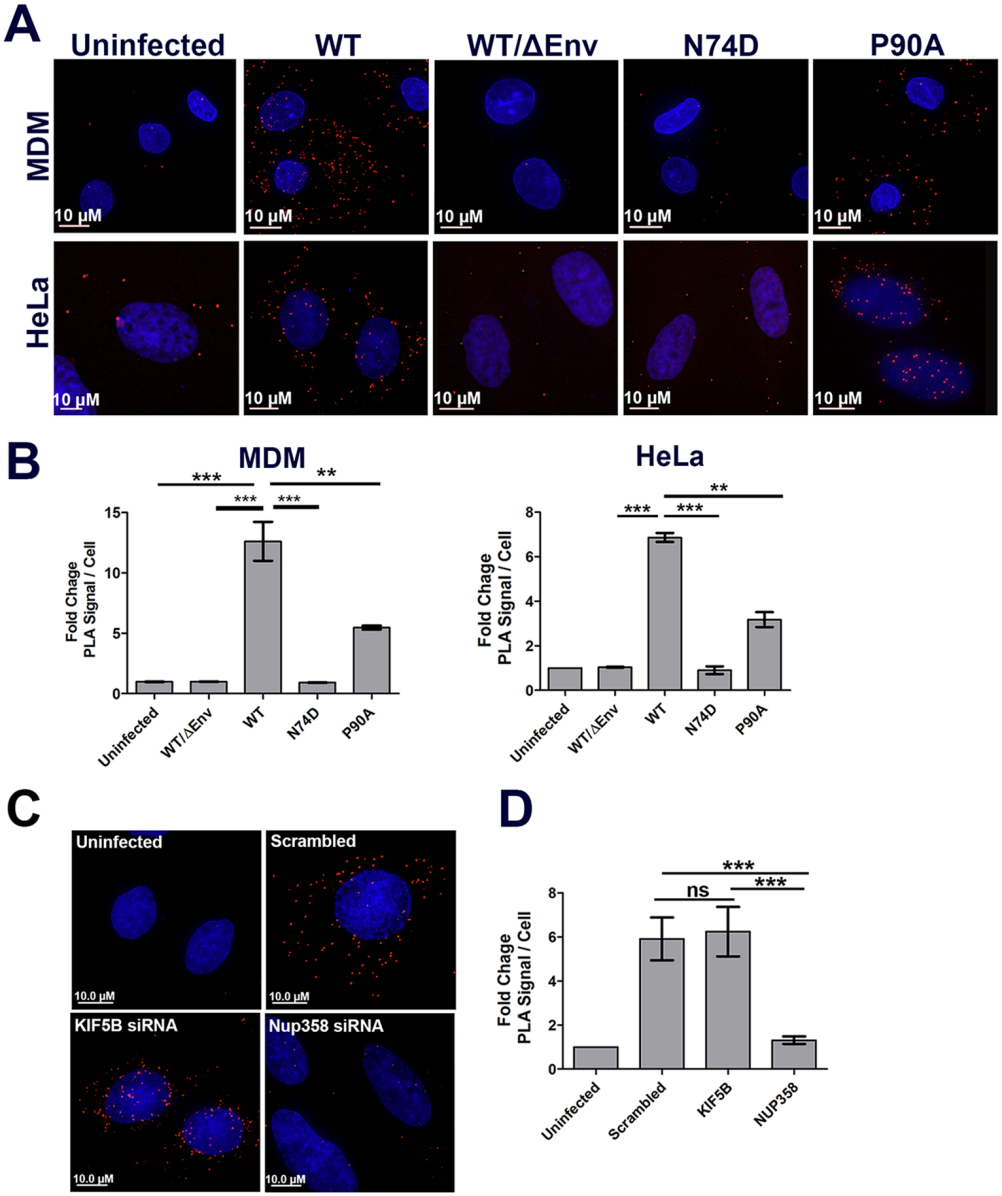
Nup358 associates with HIV-1 cores in the cytoplasm during infection. **(A)** MDM or HeLa cells were synchronously infected HIV-1 GFP pseudotyped with VSV-g bearing either the wildtype (WT) CA or N74D and P90A CA mutants(MDMs MOI 0.3, HeLa MOI 0.6).Cells fixed at 3h post infection, incubated with primary antibodies to Nup358 and HIV-1 CA, followed by secondary antibodies specific for these antibodies conjugated to the PLUS and MINUS PLA oligonucleotides. Each red fluorescent puncta represents a positive PLA signal generated by the interaction of PLUS and MINUS oligonucleotides bound to secondary antibodies. **(B)** Quantification of PLA signal in MDMs and HeLa cells, as measured by the average fold increase in PLA signal, relative to uninfected control, in three independent experiments.**(C)** HeLa cells were transfected with siRNA’s targeting KIF5B, Nup358 or scrambled control siRNA and synchronously infected with R7ΔEnvGFPpseudotyped with VSV-g (MOI 0.6) 96 hours following siRNA transfection. Cells fixed at 3h post infection and PLA assay performed as described in (A). **(D)** Quantification of PLA signal in the siRNA knockdown cells as in (B). 20 or more cells were analyzed in each experiment. Error bars represent the SEM(**p<0.01, *p<0.05, ns = not significant). Data is representative of three or more independent experiments.

To further validate this observation, we performed similar experiments as above but employed the proximity ligation assay (PLA). This assay utilizes species specific secondary antibody probes conjugated to complementary oligonucleotides. When two such antibodies are in close proximity (<30-40nm), the complementary strands can be ligated and amplified and detected as bright fluorescent puncta, thus measuring protein-protein interaction with high specificity and sensitivity[40,41,42,43,44]. In a PLA using primary antibodies to CA and NUP358, PLA puncta were readily detected in the cytoplasm of MDMs and HeLa cells infected with WT virus, while a smaller number of puncta were observed following infection with WT/ΔEnv virus. The number of puncta quantified following WT/ΔEnv infection was similar to the number of background puncta in uninfected control cells (Fig 6A and 6B). The N74D mutant also failed to induce PLA puncta following infection (Fig 6A and 6B), consistent with the lack of colocalization observed by immunofluorescence analysis (Fig 5A and 5B). However, we did observe that infection with the P90A mutant induced more PLA puncta than the N74D mutant and other controls (Fig 6A and 6B), although the number of puncta induced by this mutant was consistently lower than WT in MDMs and HeLa cells (Fig 6B). Collectively, these experiments demonstrate a specific association between WT viral cores and Nup358 in the cytoplasm of target cells that is dependent on the hydrophobic pocket disrupted by the N74D mutation and facilitated by the Cyp binding loop disrupted by the P90A mutation.

We next used the NUP358/CA PLA assay to determine if KIF5B was necessary to promote the interaction between CA and NUP358. Infection of HeLa cells transfected with control or KIF5B specific siRNA did not influence the number of PLA puncta observed following infection with WT virus (Fig 6C and 6D). However, the PLA puncta were predominantly localized to a perinuclear region following KIF5B depletion. Finally, siRNA depletion of NUP358 reduced the number of PLA puncta observed, demonstrating the specificity of the assay. These data demonstrate that engagement of NUP358 by HIV cores occurs independently of KIF5B.

### Anterograde trafficking of HIV-1 cores from the nuclear envelope

**Fig 7:**
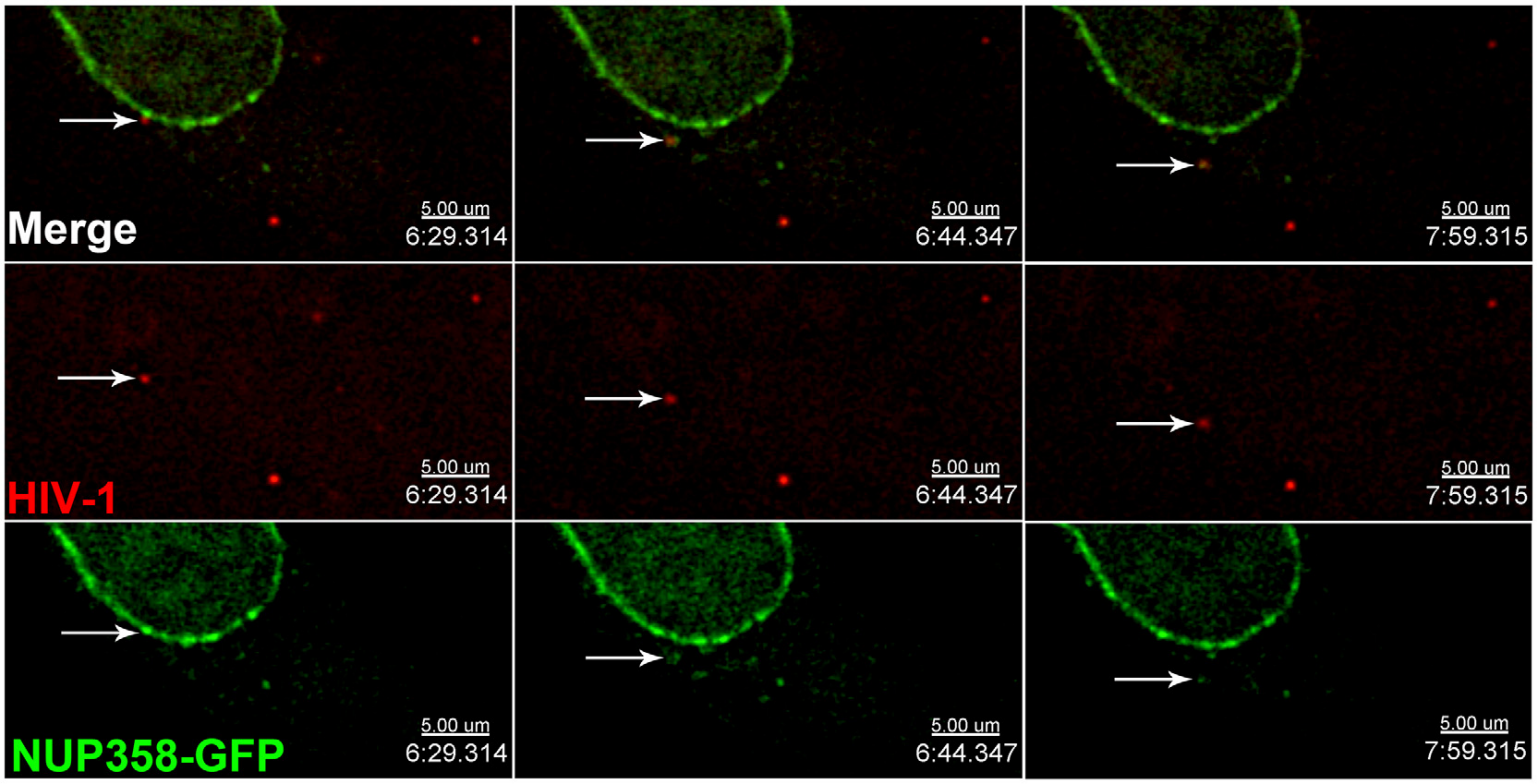
Anterograde trafficking of HIV-1 cores during infection. HeLa cells transfected with a NUP358-GFP expressing BAF were synchronously infected with GIR labelled HIV-1 viral particles (MOI 0.3). 2 hours after infection, cells were imaged every 15 seconds for 10 minutes. Shown are individual z-planes showing a virus observed to traffic away from the nucleus during the acquisition period while associated with NUP358-GFP.

The data above demonstrates that HIV-1 cores can induce the cytoplasmic accumulation of NUP358, and this relocalization of NUP358 is prevented by KIF5B depletion. Taken together with the observation that KIF5B depletion results in the perinuclear accumulation of viral cores, this suggests that the viral core reaches the nuclear pore and is subsequently trafficked away from the NPC by KIF5B. To test this hypothesis, we labeled synchronously infected these cells with HIV-1 virions labeled with a Gag-Integrase-Ruby (GIR) construct. This Ruby variant of a recently described GFP construct [45], contains a protease cleavage site between Gag and Integrase, such that viral maturation results in the liberation of Integrase-Ruby when the construct is expressed in virus producing cells. We used GIR labeled HIV-1 to infect HeLa cells transfected cells with a bacterial artificial chromosome expressing NUP358-GFP [46]. In cells, this NUP358-GFP construct localized to the nuclear envelope and to cytoplasmic puncta which were present in the presence or absence of HIV-1 infection. Live cell imaging was performed 1–3 hours following infection. At this time following infection, numerous examples of viruses associated with the nuclear envelope could be observed. Such viruses were frequently observed to traffic along the periphery of the nucleus, consistent with a previous report [47]. Viruses exhibiting this behavior could frequently be observed to become displaced from the nuclear envelope. As shown in S1 Movie, the indicated virus can be observed to traffic away from the nucleus and ultimately return to the area of the nuclear envelope during a 10-minute acquisition period (S1 Movie). As seen in this movie, we did not detect sustained trafficking of NUP358-GFP with HIV-1, although it is possible that the imaging conditions required for live cell imaging were insufficient to detect small amounts of NUP358-GFP associated with HIV-1 cores in these situations. We did, however, frequently observe cytoplasmic associations between HIV-1 and NUP358-GFP that are consistent with the displacement of NUP358-GFP from the nuclear envelope (Fig 7, S2 Movie). In the sequence provided, an accumulation of NUP358-GFP appears and colocalizes with a viral particle that was associated with the nuclear envelope in the previous frame. As apparent in the movie, this association does not appear uniformly stable throughout the acquisition period. However, some amount of NUP358-GFP signal remains present on this particle 90 seconds after it becomes displaced from the nuclear envelope. These data are consistent with the displacement of NUP358-GFP from the nuclear pore. However, we cannot exclude the possibility that the colocalization observed was between HIV-1 and one of the numerous preexisting, cytoplasmic accumulation of NUP358 in these cells, which could be seen trafficking considerable distances during the 15 second acquisition intervals. However, these experiments do demonstrate anterograde trafficking of HIV-1 from the nuclear envelope and frequent but transient periods of colocalization between HIV-1 and cytoplasmic NUP358-GFP.

#### NUP358 association with HIV-1 cores is CPSF6 dependent and Cyclophilin A independent

**Fig 8:**
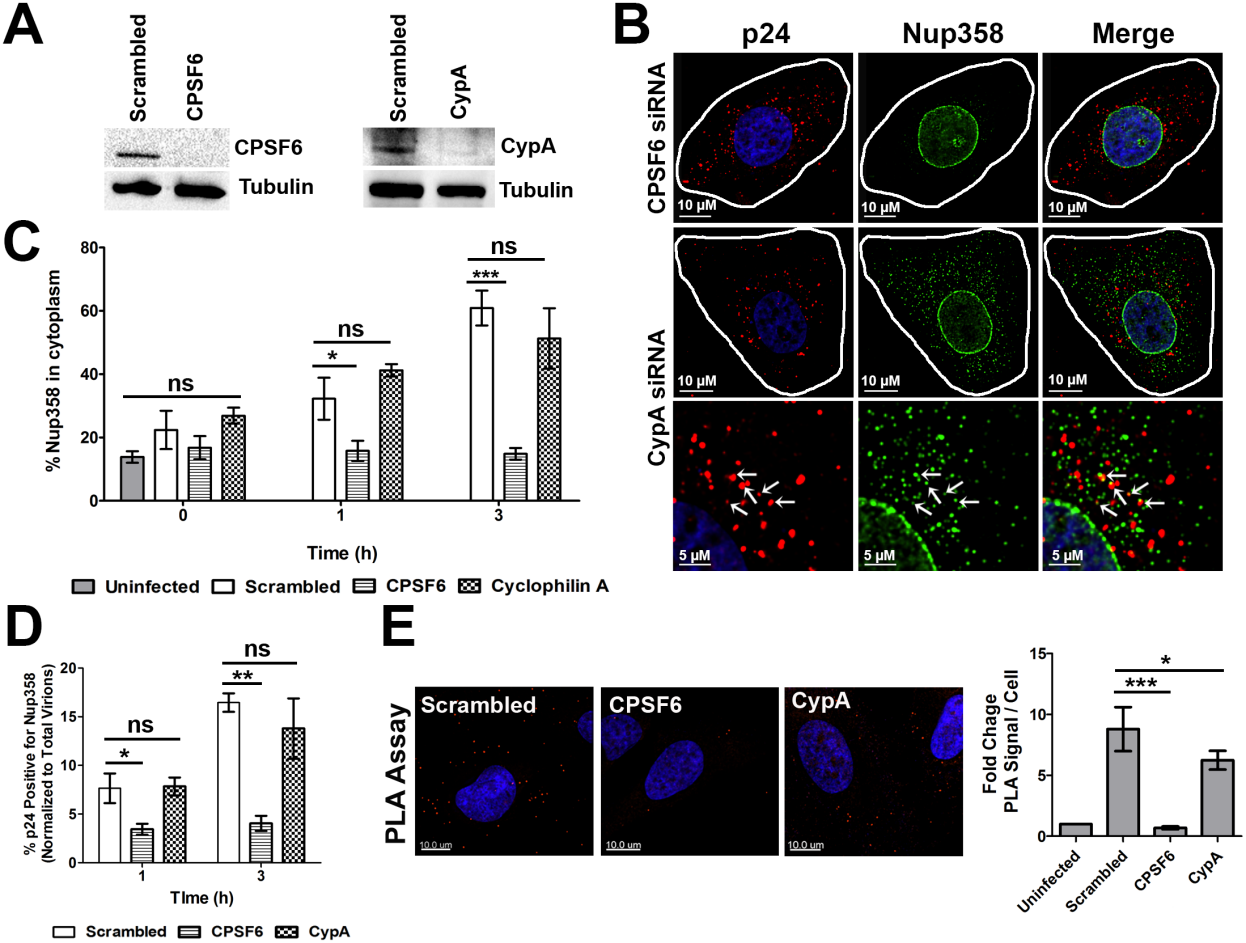
Nup358 association with HIV-1 cores is CPSF6 dependent and Cyclophilin A independent. HeLa cells transfected with scrambled siRNA or siRNA specific for CPSF6 or CypA.**(A)**Western blot for CPSF6 or CypA 96h following siRNA transfection. **(B)**siRNA depleted cells were synchronously infected with VSVg-R7ΔEnvGFP(MOI 0.6). Cells were fixed 0, 1 and 3h post infection stained for HIV-1 capsid protein p24 (red) and NUP358 (green).Depicted a representative image at 3h post infection. Nup358 and CA colocalization following CypA depletion shown in bottom panel. **(C)**The fraction of Nup358 signal in the cytoplasm at the indicated time PI. **(D)** Quantification of CA and Nup358 signal colocalization in the siRNA depleted cells. **(E)** PLA of Nup358 and CA, performed on the siRNA depleted cells fixed 3 hours following synchronous infection, and quantification of average fold increase in PLA signal. 20 or more cells were analyzed in each experiment. Error bars represent the SEM (**p<0.01, *p<0.05, ns = not significant). Data is representative of three or more independent experiments.

As the N74D and P90A mutations are known to disrupt interactions with CPSF6 and Cyclophilin A (CypA), respectively [3,18,23,24,25,26], we determined the degree to which each factor was required for the interactions observed between HIV-1 viral cores and NUP358. Following siRNA mediated knockdown in HeLa cells, the ability of HIV-1 to induce the redistribution of NUP358 to the cytoplasm as assessed. We observe that CPSF6 depletion, but not CypA depletion, prevented the redistribution of Nup358 to the cytoplasm (Fig 8A, 8B and 8C). Correspondingly, cytoplasmic colocalization of NUP358 and CA was observed in cells depleted of CypA (Fig 8B and 8D), but not in CPSF6 depleted cells (Fig 8D). Similarly, in the PLA assay, CPSF6, but not CypA depletion, inhibited the formation of PLA puncta generated using antibodies to Nup358 and CA (Fig 8E). Similarly, treatment of cells with CsA, an inhibitor of CypA, did not affect Nup358 relocalization or cytoplasmic colocalization with CA (S5 Fig).

## Discussion

In this study, we demonstrate that HIV-1 infection induces the KIF5B dependent relocalization of Nup358. This relocalization of Nup358, and cytoplasmic association with HIV-1, was dependent on entry of HIV-1 into the target cell cytoplasm, as this relocalization was not observed using WT/ΔEnv HIV-1. Although the role of Nup358 in facilitating HIV-1 infection has been observed by others [12,14,16,17,18,26,27], a clear understanding of the molecular interactions occurring between CA and Nup358 during infection has not emerged. As a large, multi-domain protein associated with the cytoplasmic side of the NPC, numerous functions have been ascribed to Nup358 with respect to its role in HIV-1 infection[16,18,26,27]. Two studies have supported a role for Nup358 driving HIV-1 uncoating via interaction with the Cyp homology domain in Nup358 [18,26]. However, another study which demonstrated that the Cyp homology domain is not required for the Nup358 dependent enhancement of HIV-1 infection suggests that the role of Nup358 in HIV-1 infection cannot be fully explained by the Cyp homology domain [27]. To understand the determinants in Nup358 required for the interaction with HIV-1 cores, we exploited the measurable association between Nup358 and HIV-1 cores in the cytoplasm, and CA mutants which disrupt putative Nup358 binding surfaces. Specifically, we observed that mutations which disrupt the hydrophobic binding pocket in assembled CA (N74D) and mutations which disrupt the conserved Cyp binding loop on CA (P90A) both perturb the relocalization of Nup358 induced by HIV-1 infection (Fig 4) and the association of CA and Nup358 during infection (Figs 5 and 6). The association of CA and Nup358 in the cytoplasm was measured in two distinct but related methods. When the intensity of Nup358 staining associated with individual viral cores was measured, neither the N74D or P90A mutant exhibited significantly more colocalization with Nup358 than WT/ΔEnv control virus (Fig 5), suggesting that both CA epitopes are required for Nup358 association. However, when the interaction between CA and Nup358 was measured by PLA, we observed a significant degree of interaction between P90A CA and Nup358, while the N74D mutant did not induce significantly more puncta than was observed following WT/ΔEnv or mock infection (Fig 6). This suggests that although the association between CA and Nup358 may be facilitated by both the hydrophobic binding pocket and Cyp binding loop of CA, the hydrophobic pocket is required for this interaction. Of note, the PLA, by its nature, detects the frequency but not the magnitude of interactions between the viral core and Nup358, as the puncta identified are generated by enzymatic amplification of nucleotides when individual secondary antibodies are in close enough proximity to enable the reaction. As such, the PLA sensitively detects the interaction between two proteins, rather than the degree of association. Despite these methodological differences, both methods demonstrated that the N74D mutation prevented the association of Nup358 with viral cores. Differences in the colocalization analysis and PLA analysis suggest that the P90A mutation reduces, but does not eliminate, the binding of Nup358 to CA. Multivalent binding of Nup358 and the viral core may help to reconcile previously discordant observations regarding the role of the Cyp homology domain in infection [18,26,27]. We also observe that CPSF6 depletion similarly prevents the redistribution of NUP358 to the cytoplasm and HIV-1 association. This suggests that CPSF6 somehow facilitates the interaction between HIV-1 and NUP358, consistent with a role for CPSF6 in HIV-1 nuclear import observed in other imaging based approaches [48,49]. CPSF6 is known to bind to the hydrophobic pocket, formed between adjacent CA monomers, and this pocket is known to be disrupted by the N74D mutation [22,23,24,25,50]. The similarity in the results obtained following CPSF6 depletion and using the N74D mutant suggest a common mechanism. However, given the large number of hydrophobic pockets present in the viral core, it remains unclear if CPSF6 acts as an adaptor linking the viral core and NUP358, or if CPSF6 engagement somehow facilitates the ability of NUP358 to bind the core in similar, adjacent hydrophobic pockets present on the core.

**Fig 9:**
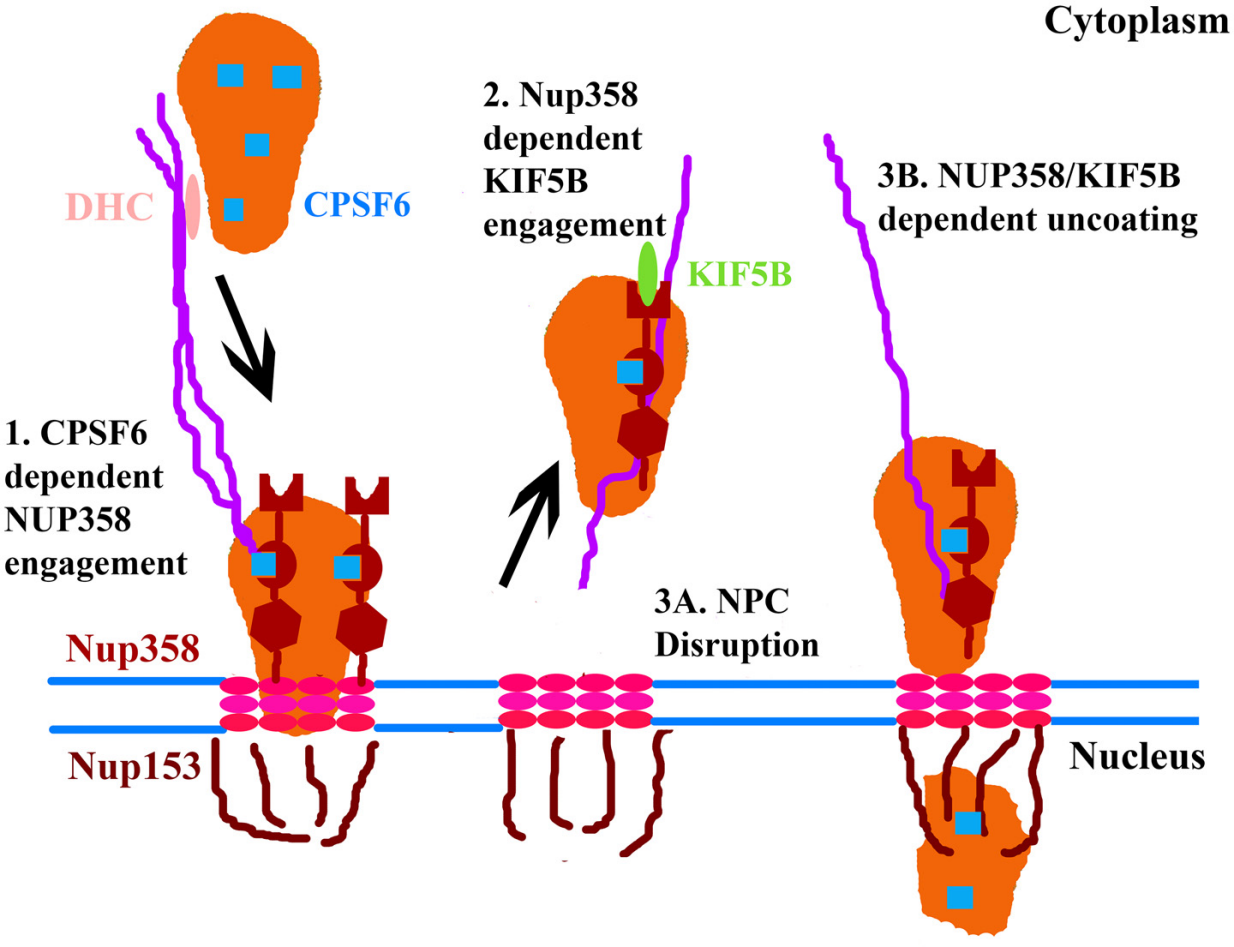
Model of KIF5B mediated nuclear import of HIV-1. **(1)** During the early steps of infection, CPSF6 binding to the viral core occurs. This interaction between CPSF6 (blue squares) and the viral core and cytoplasmic dynein heavy chain (DHC) mediated trafficking along microtubules is necessary to facilitate an interaction between NUP358 and the core. **(2)**Core engagement by Nup358 is necessary to trigger the KIF5B mediated trafficking of both the core and Nup358 away from the nuclear pore. There are two potential mechanisms by which this KIF5B trafficking facilitates the nuclear import of the viral genome. **(3A)**First, NPC disruption, induced through KIF5B mediated dislocation of Nup358 away from the viral core, may facilitate the ability of the PIC to enter the nucleus by enhancing nuclear permeability, as previously demonstrated in the case of adenovirus infection [52]. **(3B)**Second, cytoplasmic uncoating, cooperatively mediated by NUP358 and KIF5B in the cytoplasm, may ultimately reduce the size of the PIC to dimensions compatible with translocation across the nuclear pore.

One limitation in imaging based studies of viral trafficking, including this one, is that such methods analyze bulk populations of viruses at early times following infection, and therefore analyze many viral particles which are not destined to lead to productive infection. However, in this study, the phenotypes observed using imaging based approaches were in good agreement with qPCR based detection of reverse transcription (Late RT), nuclear import (2 LTR-circles) and infection. We observe that infection and nuclear import of WT HIV-1 is perturbed by KIF5B knockdown, consistent with recent observations by us and others [28,29]. Critically, however, we also observe here that the N74D and P90A mutants are not similarly affected by KIF5B depletion (Fig 3). Notably, nuclear entry of the N74D and P90A virus has previously been shown to be Nup358 independent [18,20]. The observation that these mutants are similarly not dependent on KIF5B for nuclear import and infection suggests that KIF5B and Nup358 act cooperatively to facilitate nuclear entry of the viral complex. In considering the mechanisms by which NUP358 and KIF5B might mediate this effect, two aspects of infection should be considered. First, the intact HIV-1 core is too large, by half, to fit through the nuclear pore [8,9,10,11], making it very unlikely that an intact core can translocate through an intact nuclear pore. However, numerous studies have recently observed intranuclear HIV-1 complexes containing CA [45,48,49], which strongly suggests that some CA must remain associated with the viral genome during nuclear import. As such, there are two mechanisms by which KI5B and Nup358 may promote the nuclear entry of the viral genome during infection (Fig 9). First, cooperative core uncoating, as we measured using an in situ uncoating assay (Fig 1), may reorganize the CA associated with the viral genome to achieve a complex with dimensions capable of translocating through the nuclear pore. Alternatively, KIF5B mediated displacement of NUP358 from the NPC may disrupt the NPC in such a way that the ability of the viral core to traverse the nuclear pore is increased. This would be consistent with a recent study by Chin and coworkers, who observed that association with CSPF6 enhanced nuclear entry and potentiates the ability of the viral genome to traffic deeper into the nucleus[48]. Taken together with the observation that KIF5B depletion inhibits nuclear import (Fig 3)[28,29], and the finding of others that Nup358 and CPSF6 depletion alters the genomic distribution of HIV-1 integration sites [18,51], we speculate that the ability of the viral core to induce the translocation of Nup358 away from the NPC in a CPSF6 dependent fashion may alter the NPC environment in a way that allows the nuclear translocation of larger cargoes, thus facilitating the deeper nuclear penetration of viral genomes, as observed by Chin et al. Such a model might also explain other studies which seem to observe alternate pathways of HIV-1 nuclear import [18,20](Fig 8). In that regard, it is notable that studies examining the uncoating and nuclear entry of adenovirus have reported that a Kinesin-1 dependent, Nup358 dependent mechanism mediates the uncoating of adenovirus at the NPC [52]. This study observed that anterograde trafficking by Kinesin-1 induced the gross disruption of the NPC, such that the nuclear entry of large molecules above the diffusion barrier was facilitated by adenovirus infection [52]. In our study, we were not able to demonstrate a similar disruption of the NPC induced by HIV-1 infection. However, these negative data do not preclude a similar NPC disruption from occurring during HIV-1 infection, as the study demonstrating NPC disruption at a cellular level used a much higher MOI than was used in our studies [52].

Our analysis into the cellular determinants which are required for this interaction between Nup358 and CA revealed that CPSF6 is required for this association, while CypA is not required for this interaction (Fig 7).

Understanding how KIF5B and Nup358 cooperatively coordinate the trafficking, uncoating and nuclear import of the viral genome may provide opportunities for therapeutic interventions expected to induce an antiviral response when this process is disrupted. In support of this possibility, Rasaiyaah and coworkers have observed that the same N74D and P90A mutants which we observe fail to associate with Nup358 (Figs 5 and 6) and enter the nucleus independently of KIF5B (Fig 3) also activate cytoplasmic host cell sensors and initiate interferon response [5]. Taken together, this suggests that NUP358 association is necessary to avoid activation of innate sensors by HIV-1 during infection. A better understanding of the cytosolic interactions which allow HIV-1 uncoating and nuclear import to occur without activation of these sensors may provide opportunities to leverage the innate and adaptive immune response to HIV-1, in the context of treatment or vaccine development.

## Supporting Information

S1 Fig siRNA depletion of KIF5B or NUP358 does not affect viral fusion. The relative fusion, measured as the percent of GFP-Vpr+ virions retaining the S15-mCherry membrane label, is shown for each time point. % fusion in HeLa cells treated with Nup358 **(A)**, KIF5B **(B)** and NUP358+5B siRNA **(C)** calculated from experiments described in Fig 1. The number of viral particles analyzed in Fig 1 represents the fraction of particles lacking S15 (fused) in the graphs above. Data is representative of three or more independent experiments.
(TIF)

S2 Fig Nup358 relocalization in cells infected with MLV. **(A)** NUP358 staining in uninfected HeLa cells and cells depleted of Nup358 using siRNA.**(B)**HeLa cells were synchronously infected with equal titers of VSVg pseudotyped R7ΔEnvGFP and B-MLV pseudotyped. Cells were fixed 0, 1 and 3h (shown) post infection and stained for Nup358. **(C)** The fraction of Nup358 signal in the cytoplasm at the indicated time PI.20 or more cells were analyzed in each sample. Error bars represent the SEM of three independent experiments. (***p<0.001, *p<0.05, ns = not significant). Data is representative of three or more independent experiments.
(TIF)

S3 Fig HIV-1 infection does not induce any change in NUP153 localization. **(A)**Nup153 staining in uninfected HeLa cells.**(B)**HeLa cells were synchronously infected with VSVg pseudotyped HIV-1 reporter virus (MOI 0.6) bearing either the wildtype (WT) CA or N74D and P90A CA mutants. Cells were fixed at 0, 1 or 3h (shown) post infection and stained for Nup153 (green).**(C)** Quantification of CA and Nup153 signal colocalization. **(D)**The fraction of Nup153 signal in the perinuclear and cytoplasm at the indicated time PI, measured as in 2C. 20 or more cells were analyzed in each sample. Error bars represent the SEM of three independent experiments. (ns = not significant). Data is representative of three or more independent experiments.
(TIF)

S4 Fig Capsid and Nup358 staining in cells infected with capsid mutants N74D and P90A. MDMs were synchronously infected with HIV-1 GFP pseudotyped with VSV-g bearing either the N74D or P90A CA mutants. Cells fixed 1 and 3h post infection and stained for viral capsid protein p24 (red) and Nup358 (green). Depicted a representative image at 3h post infection and an enlarged section of the same image. Data is representative of three or more independent experiments.
(TIF)

S5 Fig CsA treatment does not affect Nup358 association with HIV-1 cores. **(A)** MDM and HeLa cells subjected to synchronized infection with VSVg pseudotyped R7ΔEnvGFP(MDM MOI 0.3 and HeLa MOI 0.6) in the presence or absence of 2.5 μM cyclosporin A (CsA). Cells fixed at 0,1 or 3h (shown) post infection and stained for p24 (red) and Nup358 (green). **(B)** PLA assay performed on CsA treated MDM and HeLa cells3h post synchronous infection. **(C,D,E)**Quantification of the fraction of Nup358 signal in the cytoplasm at the indicated time PI **(C)**, Quantification of CA and Nup358 signal colocalization **(D)**, Quantification of the average fold increase in PLA signal **(E)** in MDMs. **(F,G,H)**Similar quantification as above in HeLa cells. 20 or more cells were analyzed in each sample. Error bars represent the SEM of three independent experiments. (ns = not significant). Data is representative of three or more independent experiments.
(TIF)

S1 Movie Anterograde trafficking of HIV-1 cores during infection. HeLa cells transfected with a NUP358-GFP expressing BAF were synchronously infected with GIR labeled HIV-1 viral particles (MOI 0.3). 2 hours after infection, cells were imaged every 15 seconds for 10 minutes. Shown are individual z-planes showing a virus observed to traffic away from the nucleus and ultimately return to the area of the nuclear envelope during the acquisition period. The arrow in the first frame indicates the viral particle exhibiting the behavior described in the text.
(MOV)

S2 Movie Anterograde trafficking of HIV-1 cores during infection. HeLa cells transfected with a NUP358-GFP expressing BAF were synchronously infected with GIR labeled HIV-1 viral particles (MOI 0.3). 2 hours after infection, cells were imaged every 15 seconds for 10 minutes. Shown are individual z-planes showing a virus observed to traffic away from the nucleus during the acquisition period while associated with NUP358-GFP. The arrow in the first frame indicates the viral particle exhibiting the behavior described in the text.
(MOV)
