# Supplementary figures and images for: KIF5B and Nup358 Cooperatively Mediate the Nuclear Import of HIV-1 during Infection
Source: PLoS Pathog. 2016 Jun 21;12(6):e1005700. doi: 10.1371/journal.ppat.1005700 (PMC4915687; doi:10.1371/journal.ppat.1005700)

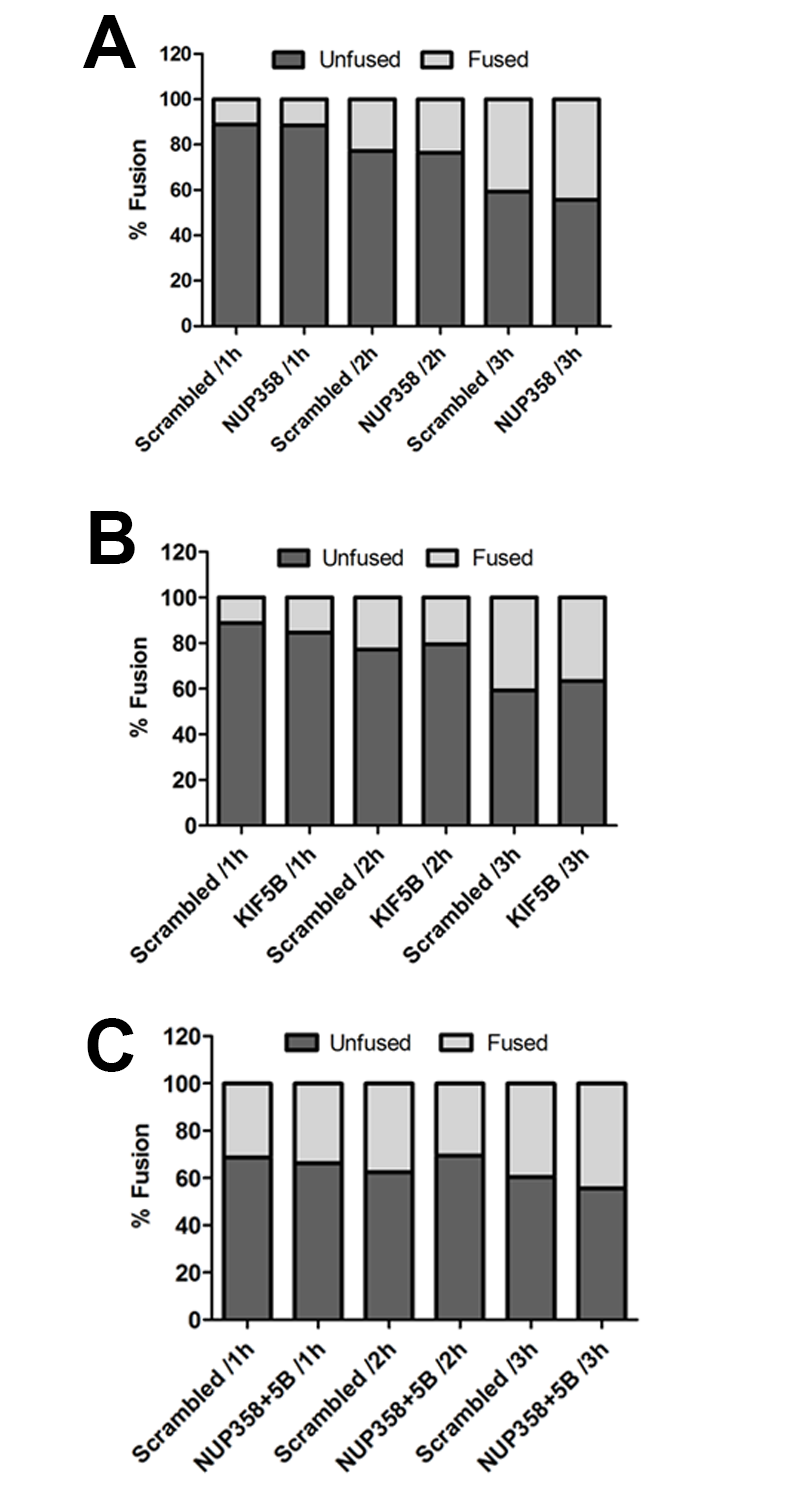

Supplement: S1 Fig — The relative fusion, measured as the percent of GFP-Vpr+ virions retaining the S15-mCherry membrane label, is shown for each time point. % fusion in HeLa cells treated with Nup358 (A), KIF5B (B) and NUP358+5B siRNA (C) calculated from experiments described in Fig 1. The number of viral particles analyzed in Fig 1 represents the fraction of particles lacking S15 (fused) in the graphs above. Data is representative of three or more independent experiments. (TIF) [file ppat.1005700.s001.tif]

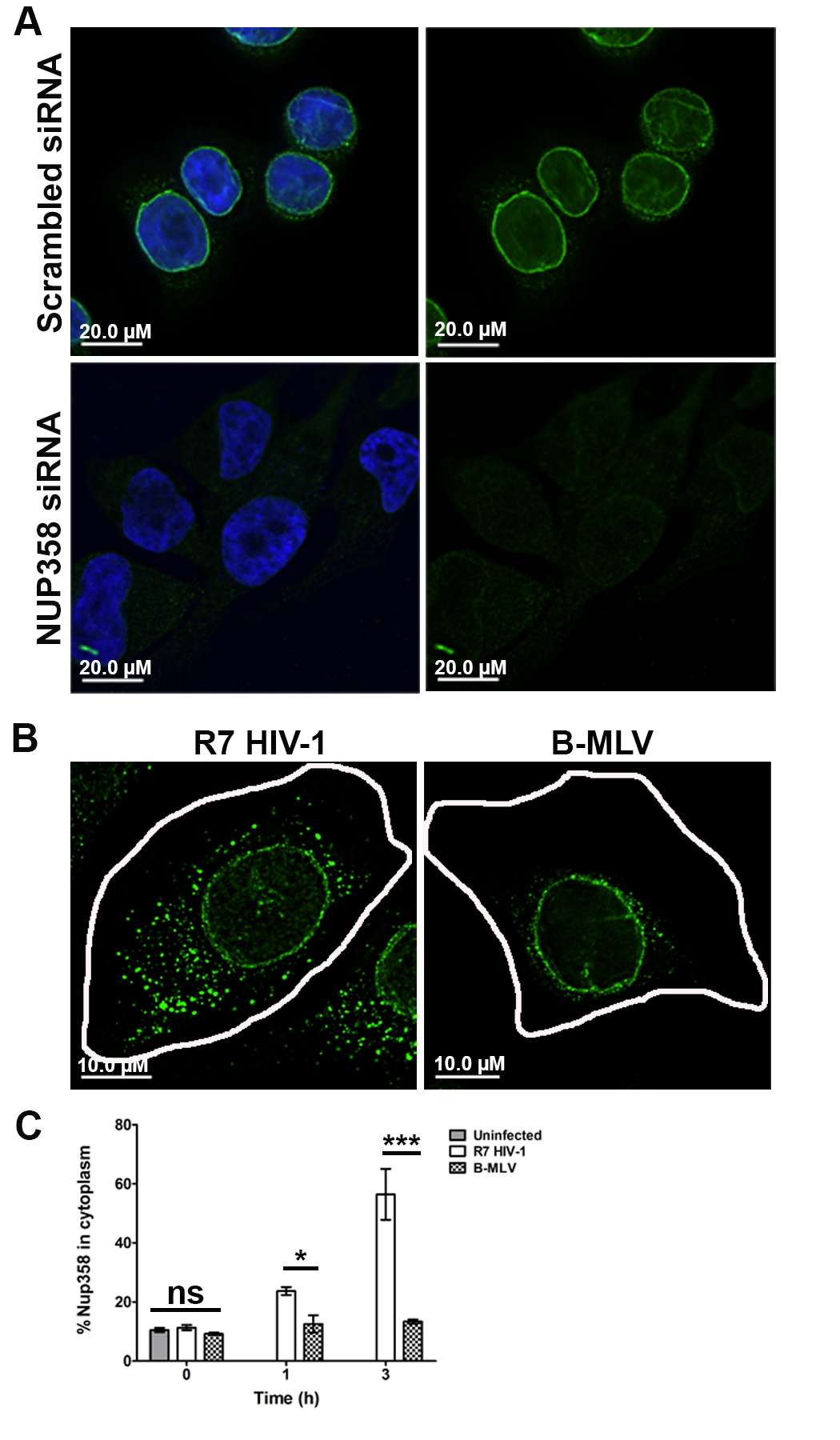

Supplement: S2 Fig — (A) NUP358 staining in uninfected HeLa cells and cells depleted of Nup358 using siRNA.(B)HeLa cells were synchronously infected with equal titers of VSVg pseudotyped R7ΔEnvGFP and B-MLV pseudotyped. Cells were fixed 0, 1 and 3h (shown) post infection and stained for Nup358. (C) The fraction of Nup358 signal in the cytoplasm at the indicated time PI.20 or more cells were analyzed in each sample. Error bars represent the SEM of three independent experiments. (***p<0.001, *p<0.05, ns = not significant). Data is representative of three or more independent experiments. (TIF) [file ppat.1005700.s002.tif]

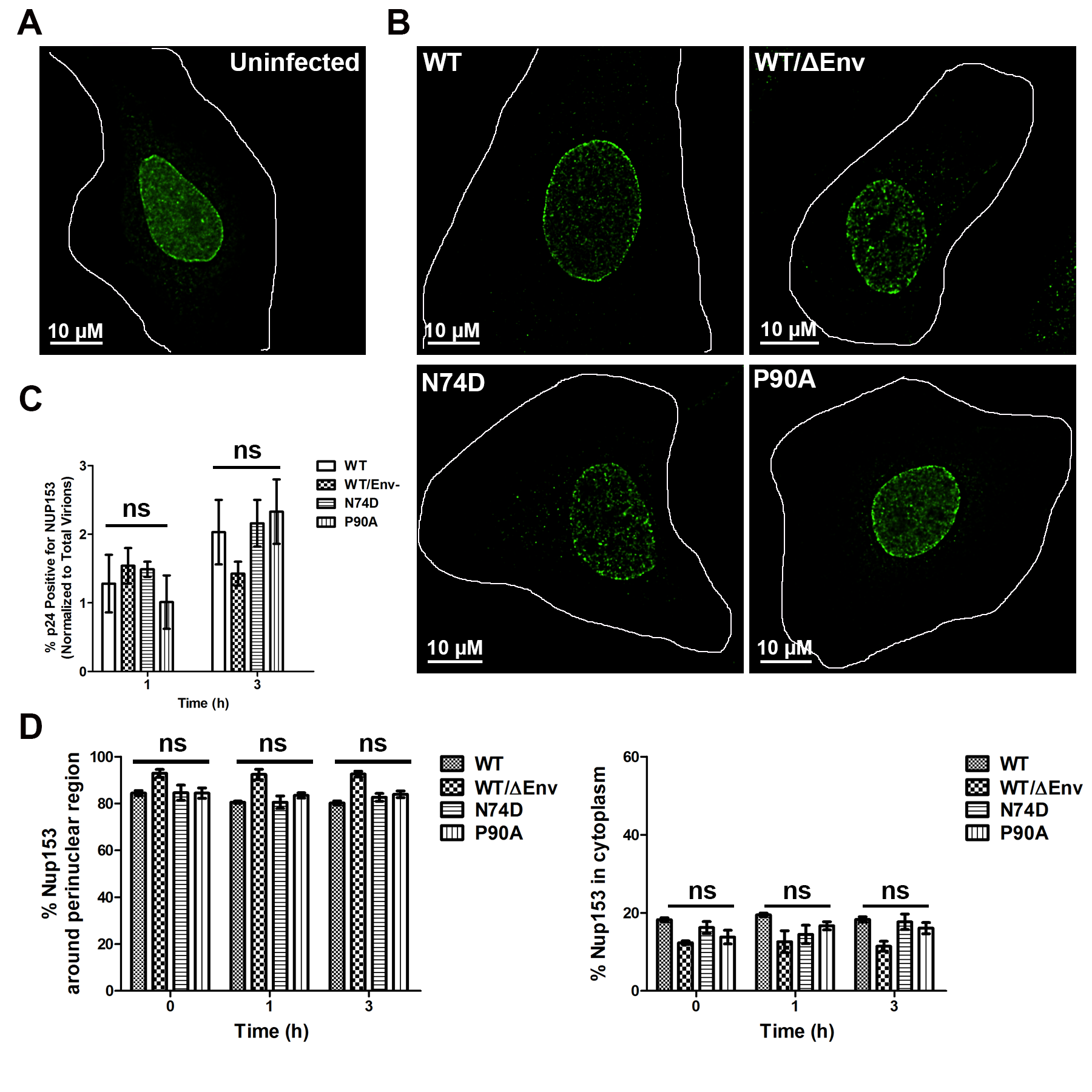

Supplement: S3 Fig — (A)Nup153 staining in uninfected HeLa cells.(B)HeLa cells were synchronously infected with VSVg pseudotyped HIV-1 reporter virus (MOI 0.6) bearing either the wildtype (WT) CA or N74D and P90A CA mutants. Cells were fixed at 0, 1 or 3h (shown) post infection and stained for Nup153 (green).(C) Quantification of CA and Nup153 signal colocalization. (D)The fraction of Nup153 signal in the perinuclear and cytoplasm at the indicated time PI, measured as in 2C. 20 or more cells were analyzed in each sample. Error bars represent the SEM of three independent experiments. (ns = not significant). Data is representative of three or more independent experiments. (TIF) [file ppat.1005700.s003.tif]

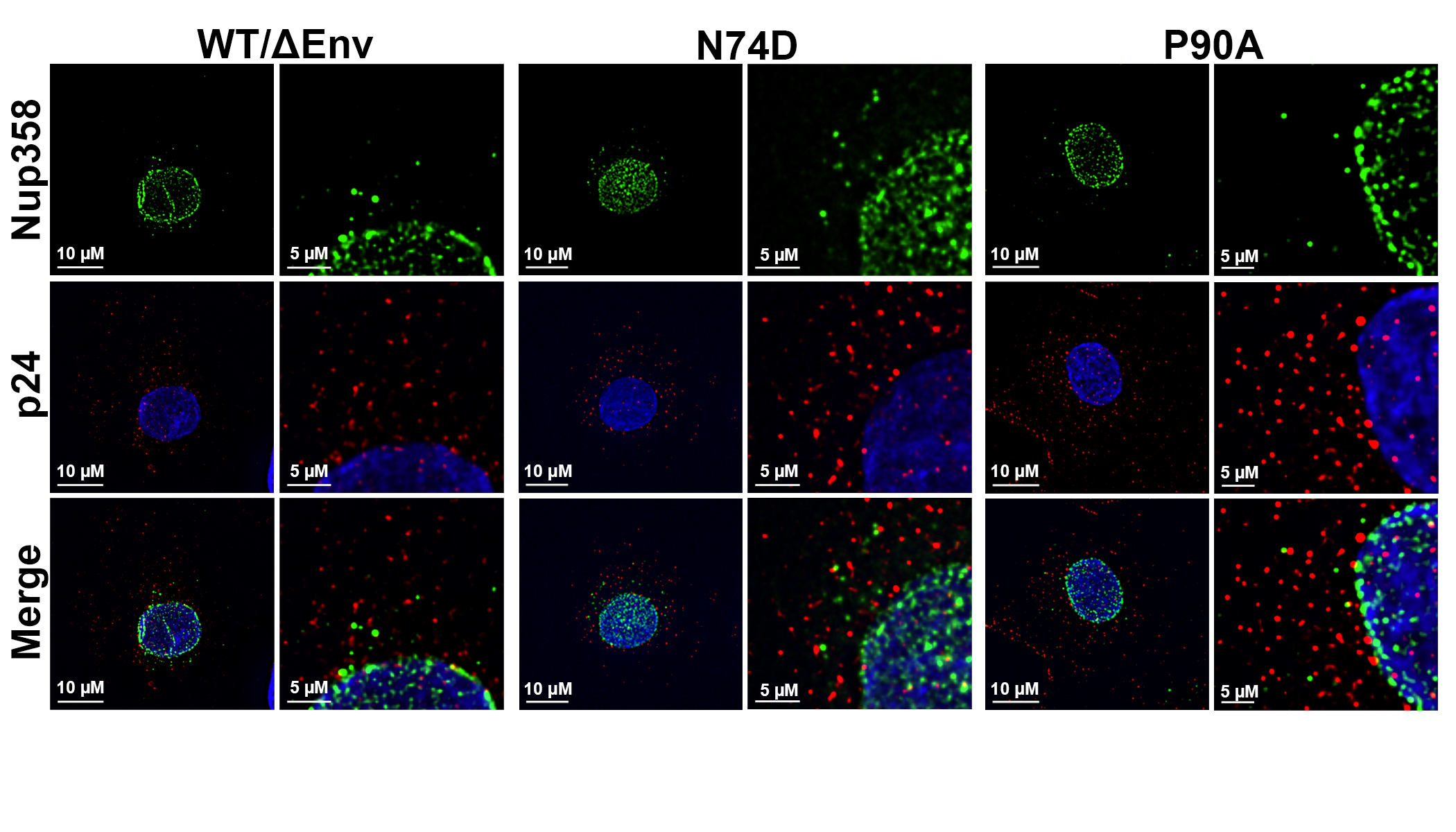

Supplement: S4 Fig — MDMs were synchronously infected with HIV-1 GFP pseudotyped with VSV-g bearing either the N74D or P90A CA mutants. Cells fixed 1 and 3h post infection and stained for viral capsid protein p24 (red) and Nup358 (green). Depicted a representative image at 3h post infection and an enlarged section of the same image. Data is representative of three or more independent experiments. (TIF) [file ppat.1005700.s004.tif]

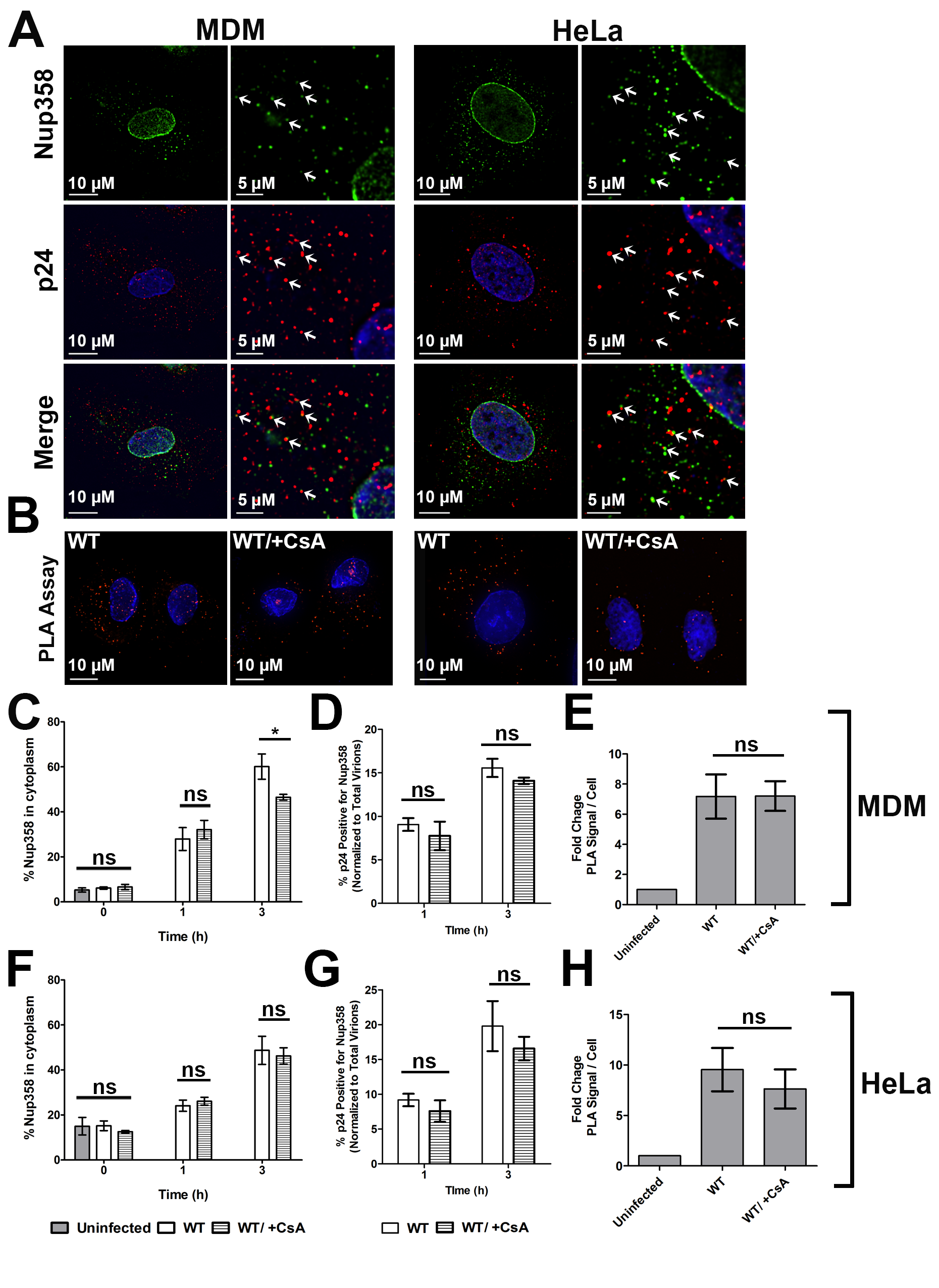

Supplement: S5 Fig — (A) MDM and HeLa cells subjected to synchronized infection with VSVg pseudotyped R7ΔEnvGFP(MDM MOI 0.3 and HeLa MOI 0.6) in the presence or absence of 2.5 μM cyclosporin A (CsA). Cells fixed at 0,1 or 3h (shown) post infection and stained for p24 (red) and Nup358 (green). (B) PLA assay performed on CsA treated MDM and HeLa cells3h post synchronous infection. (C,D,E)Quantification of the fraction of Nup358 signal in the cytoplasm at the indicated time PI (C), Quantification of CA and Nup358 signal colocalization (D), Quantification of the average fold increase in PLA signal (E) in MDMs. (F,G,H)Similar quantification as above in HeLa cells. 20 or more cells were analyzed in each sample. Error bars represent the SEM of three independent experiments. (ns = not significant). Data is representative of three or more independent experiments. (TIF) [file ppat.1005700.s005.tif]
